# Aluminium 8-Hydroxyquinolinate *N*-Oxide as a Precursor to Heterometallic Aluminium–Lanthanide Complexes

**DOI:** 10.3390/molecules29020451

**Published:** 2024-01-17

**Authors:** Elisa Gallo, Luca Bellucci, Silvia Carlotto, Gregorio Bottaro, Luca Babetto, Luca Giordano, Fabio Marchetti, Simona Samaritani, Lidia Armelao, Luca Labella

**Affiliations:** 1 Dipartimento di Chimica e Chimica Industriale and CIRCC, Università di Pisa, via Giuseppe Moruzzi 13, I-56124 Pisa, Italyluca.giordano_94@yahoo.it (L.G.);; 2 ICMATE-CNR and INSTM, Presso Dipartimento di Scienze Chimiche, Università di Padova, via Marzolo 1, I-35131 Padova, Italy; silvia.carlotto@unipd.it (S.C.);; 3 Dipartimento di Scienze Chimiche and INSTM, Università di Padova, via Marzolo 1, I-35131 Padova, Italy; 4 Dipartimento di Scienze Chimiche e Tecnologie dei Materiali (DSCTM), Consiglio Nazionale Delle Ricerche, Piazzale A. Moro 7, I-00185 Roma, Italy

**Keywords:** heterometallic complexes, lanthanides, aluminium, 8-hydroxyquinoline *N*-oxide, X-ray structure, quantum mechanical calculations

## Abstract

A reaction in anhydrous toluene between the formally unsaturated fragment [Ln(hfac)_3_] (Ln^3+^ = Eu^3+^, Gd^3+^ and Er^3+^; Hhfac = hexafluoroacetylacetone) and [Al(qNO)_3_] (HqNO = 8-hydroxyquinoline *N*-oxide), here prepared for the first time from [Al(O^t^Bu)_3_] and HqNO, affords the dinuclear heterometallic compounds [Ln(hfac)_3_Al(qNO)_3_] (Ln^3+^ = Eu^3+^, Gd^3+^ and Er^3+^) in high yields. The molecular structures of these new compounds revealed a dinuclear species with three phenolic oxygen atoms bridging the two metal atoms. While the europium and gadolinium complexes show the coordination number (CN) 9 for the lanthanide centre, in the complex featuring the smaller erbium ion, only two oxygens bridge the two metal atoms for a resulting CN of 8. The reaction of [Eu(hfac)_3_] with [Alq_3_] (Hq = 8-hydroxyquinoline) in the same conditions yields a heterometallic product of composition [Eu(hfac)_3_Alq_3_]. A recrystallization attempt from hot heptane in air produced single crystals of two different morphologies and compositions: [Eu_2_(hfac)_6_Al_2_q_4_(OH)_2_] and [Eu_2_(hfac)_6_(µ-Hq)_2_]. The latter compound can be directly prepared from [Eu(hfac)_3_] and Hq at room temperature. Quantum mechanical calculations confirm (i) the higher stability of [Eu(hfac)_3_Al(qNO)_3_] vs. the corresponding [Eu(hfac)_3_Alq_3_] and (ii) the preference of the Er complexes for the CN 8, justifying the different behaviour in terms of the Lewis acidity of the metal centre.

## 1. Introduction

**Scheme 1:**
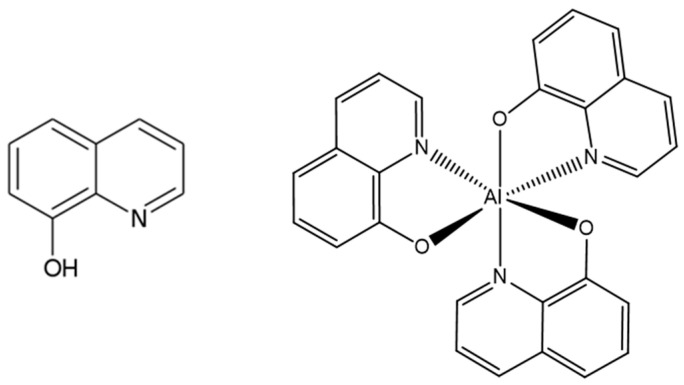
8-hydroxyquinoline (Hq) and the aluminium complex [Alq_3_].

The rational design of heterometallic compounds with a selective introduction of lanthanide and *d*-block or *p*-block metal ions into the same molecular architecture has attracted great attention in the literature [1,2,3,4,5]. The synthesis of heterometallic complexes is often challenging for the high lability of lanthanide ions, their low stereochemical preference and their tendency toward high and variable coordination numbers. Heterotopic divergent ligands containing oxygen and nitrogen donor atoms can lead to the selective binding of different metal ions, exploiting the different affinities of lanthanides and transition metal ions for *N* and *O* donors. Their use as building blocks of preformed molecular complexes, presenting hypodentate divergent ligands, may guarantee control of the synthesis of mixed metals compounds [6,7,8,9,10], with synthetic strategies normally using *d*-transition metalloligands, and less commonly, Ln-metalloligands [11,12,13,14,15,16,17,18,19,20,21]. An alternative, attractive way to prepare heterobimetallic molecular complexes exploits the reaction of formally unsaturated lanthanide fragments [Ln(diketonate)_3_] (with a diketonate carrying electron-withdrawing groups) with a mononuclear octahedral *tris*-chelate complex of a *d* or *p* metal centre acting as a Lewis base [22,23,24,25,26,27,28,29,30,31]. We recently prepared a series of europium–aluminium compounds and studied the effect of aluminium ligands on the sensitized emission of europium. Combining photoluminescence experiments with density functional theory calculations, we explained the europium emission using a model involving two noninteracting excitation paths with the ligands coordinated to aluminium and europium, respectively [32]. Octahedral mononuclear aluminium complexes with oxygen donor monoanionic chelate ligands have been found suitable to be used as Lewis bases towards heterometallic complexes. Dinuclear heterometallic complexes with the composition [Ln(hfac)_3_Al(L)_3_] (where Ln^3+^ = Eu^3+^, Gd^3+^ and Tb^3+^; Hhfac = hexafluoroacetylacetone and HL = methyl acetoacetate, Hmeac; salicylaldehyde, Hsal; and 2-hydroxynaphthaldehyde, Hnaphthal) have been characterized. In all heterometallic complexes, aluminium maintains the octahedral geometry of the precursor, while for the lanthanide, in addition to the three chelate hfac ligands, three bridging oxygen atoms of the aluminium donor fragment complete the coordination sphere. The coordination geometry of the lanthanide centre is a tricapped trigonal prism that shares one face of the octahedron of the aluminium *fac* isomer. Heterobimetallic Ln–Al complexes are relevant for catalysis [33] as luminescent materials [34] or as molecular precursors to ceramic materials [35]. Among a large number of potential precursors, the *tris* 8-hydroxiquinolinate aluminium ([Alq_3_]; Hq = 8-hydroxyquinoline, Scheme 1) attracted our attention as a stable sublimable complex, commonly used for the electroluminescent properties in OLED (organic light-emitting diode) [36,37,38,39,40,41,42]. The chemistry of [Alq_3_] and its derivatives is wide, and the functionalization of the Hq ligand can deeply impact its absorption–emission properties. This behaviour, in principle, could be exploited to develop a library of Ln–Al heterobimetallic complexes endowed with peculiar emissions or luminomagnetic properties depending on the employed lanthanoid ion. To this aim, the preliminary investigation of the experimental conditions allowing for the formation of the bimetallic complexes is fundamental.

**Scheme 2:**
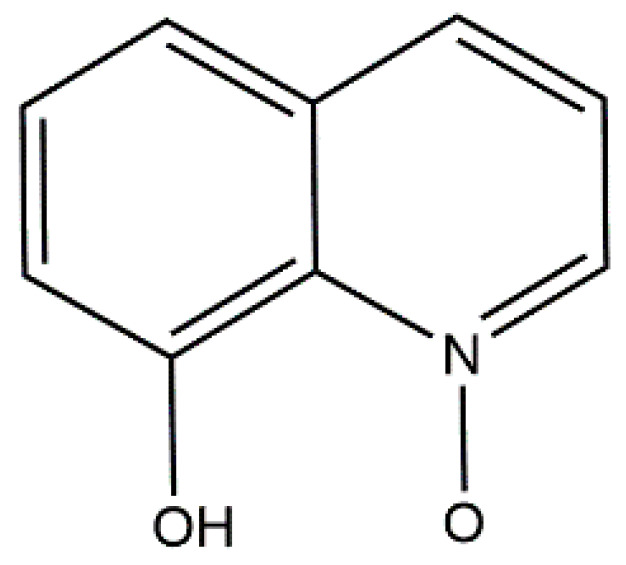
8-hydroxyquinoline *N*-oxide (HqNO).

[Alq_3_] has a mononuclear structure in an octahedral geometry, with three chelate (*N O*) asymmetric ligands. Although the *mer* isomer is thermodynamically more stable, NMR studies show the fast interconversion of the geometric isomers in solution [43]. In this work, we report the synthesis and characterization of heterometallic complexes involving the reaction of a formally unsaturated fragment [Ln(hfac)_3_] (Ln = Eu, Gd and Er) and [Al(qNO)_3_] (HqNO = 8-hydroxyquinoline *N*-oxide; Scheme 2), a new homoleptic aluminium compound with an almost-forgotten ligand in coordination chemistry. HqNO is an oxidated Hq that is able to behave, after deprotonation, as an asymmetric, monoanionic, oxygen donor, six-membered ring chelate ligand.

## 2. Results and Discussion

**Figure 1:**
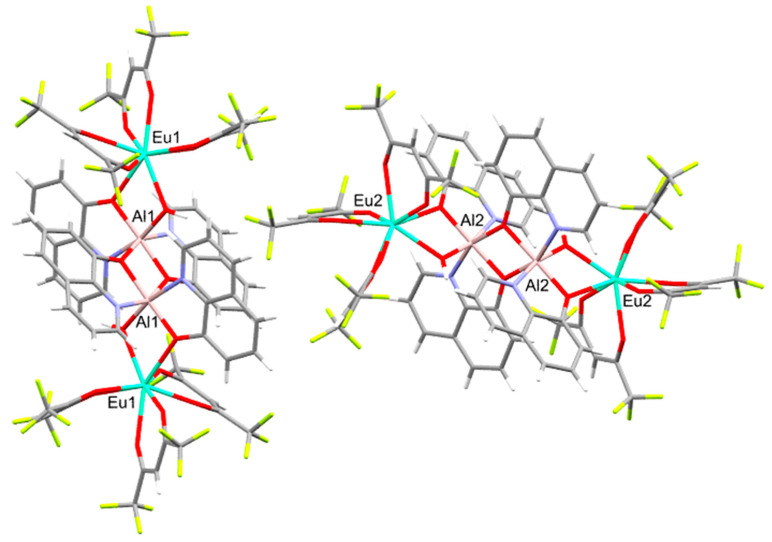
Molecular structure of the two independent molecules of [Eu_2_(hfac)_6_Al_2_q_4_(OH)_2_] (light green, europium; pink, aluminium; grey, carbon; red, oxygen; blue, nitrogen; yellow, fluorine).

The reaction between [Eu(hfac)_3_] and [Alq_3_] has been carried out in anhydrous toluene. After filtering off traces of undissolved solids, from the light-yellow solution, a product with an elemental analysis consistent with the [Eu(hfac)_3_Alq_3_] composition, **1**, was recovered at a satisfactory yield. The infrared spectrum did not show the presence of the free precursors (Figure S1). Many recrystallization attempts, aimed at obtaining suitable single crystals for a structural study using X-ray diffraction, failed. However, in a recrystallization attempt from hot heptane carried out in air, a mixture of two clearly distinguishable crystalline phases was recovered. Colourless, square-shaped crystals were mixed with large, light-yellow lozenge crystals. A single crystal X-ray diffraction study on the first kind of crystals showed the heterometallic [Eu_2_(hfac)_6_Al_2_q_4_(OH)_2_] compound, **2**. Two independent centrosymmetric molecules with the same composition are present. Both molecules present two octacoordinated europium and two hexacoordinated aluminium centres (Figure 1). The asymmetric unit consists of two half molecules, where the inversion centres are placed in the middle of the aluminium atoms.

**Figure 2:**
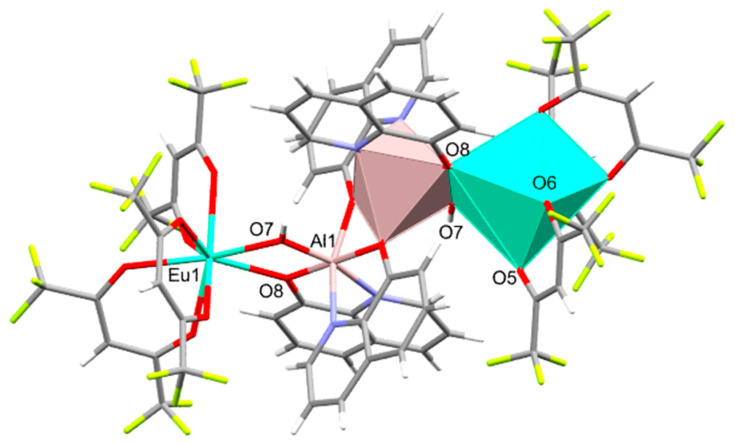
Polyhedral representation of [Eu_2_(hfac)_6_Al_2_q_4_(OH)_2_], **2** (light green, europium; pink, aluminium; grey, carbon; red, oxygen; blue, nitrogen; yellow, fluorine).

In the europium coordination sphere, six oxygen atoms of three diketonates, a bridging oxygen atom of a quinolinate and a bridging hydroxyl are present (Figure 1). Two chelate quinolinates, an oxygen atom of a quinolinate bridging the two aluminium centres and the bridging hydroxyl are present in the coordination sphere of the aluminium centre. The square antiprism of europium shares an edge (O7 O8) with the octahedral polyhedra of aluminium (Figure 2). 

In this compound, the molar ratio between the two metals used for the synthesis has been maintained, although hydrolysis has displaced a quinolinato ligand in the aluminium coordination sphere, releasing Hq. The previously reported [44] molecular structure of the heterometallic aggregate [Al_3_(Mq)_4_(HMq)(µ^3^-OH)_2_(µ-OH)_3_{Ln(hfac)_3_}_2_] (Mq = 2-methyl-8-hydroxyquinolinate; Ln = Nd, Eu and Yb), similar in some respects to our compound, has been obtained, starting from a hydrated lanthanide precursor [Ln(hfac)_3_(H_2_O)_2_] in a synthesis yielding a more hydrolysed product. 

**Figure 3:**
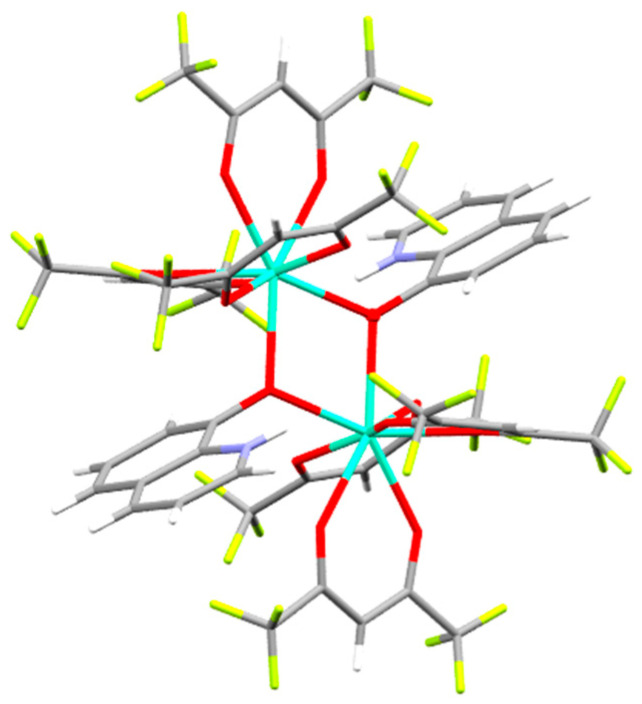
Molecular structure of [Eu_2_(hfac)_6_(µ-Hq)_2_], **3** (light green, europium; pink, aluminium; grey, carbon; red, oxygen; blue, nitrogen; yellow, fluorine).

The release of Hq can give hints on the formation of the different light-yellow crystals. The structural study carried out using X-ray diffraction identified the dinuclear europium compound [Eu_2_(hfac)_6_(µ-Hq)_2_], **3** (Figure 3). Each octacoordinated europium atom has six O atoms of three hfac and two O atoms of two Hq ligands in a distorted square antiprism geometry. The molecule is centrosymmetric, with the inversion centre being in the middle of the two oxygen atoms of the two Hq ligands. Two bridging Hq ligands adopt a zwitterionic coordination mode, with the hydrogen placed on the nitrogen atom interested in a relevant hydrogen bond with a fluorine atom of a hfac ligand. 

The cell metric closely looks like that of [Eu_2_(hfac)_6_(µ-HMq)_2_] [45], an analogous compound with a methylated quinoline prepared by reacting the green intermediate obtained from [CrCl_3_·6H_2_O], Mq and 4-hydroxybenzonitrile with [Eu(hfac)_3_(H_2_O)_2_]. 

**Scheme 3:**
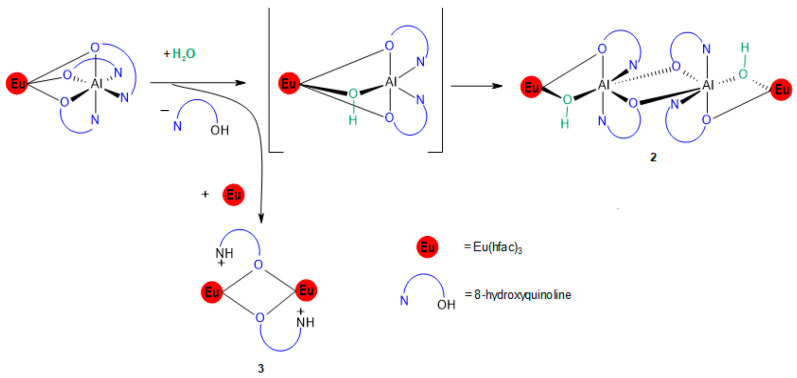
Hydrolytic path for decomposition of [Eu(hfac)_3_Alq_3_], **1**.

To rationalize our results, it is worth noting that [Alq_3_] is normally obtained from an aqueous solution [46] so that, most likely, the observed hydrolytic process concerns the heterometallic product. It appears plausible that, in the presence of water, a protic attack to a bridging quinolinato ligand in the heterometallic product displaces a quinoline that is replaced by a bridging hydroxyl group. The following structural reorganization is probably due to the preference of aluminium for an octahedral geometry (Scheme 3): 

Since [Alq_3_] is not reactive towards water, we believe that this reactivity can be favoured by the steric demands of the three quinolinates bridging the two metals. The overall encumbrance is greatly reduced in the molecular architecture of the X-ray-characterized heterometallic product. As already mentioned, the protic attack releases a quinoline, Hq, a neutral molecule, reasonably a better ligand with respect to [Alq_3_] for the formally unsaturated fragment [Eu(hfac)_3_] (Scheme 3). This point has been clarified by an independent experiment. [Eu_2_(hfac)_6_(µ-Hq)_2_] has been directly prepared from anhydrous [Eu(hfac)_3_] and Hq in toluene at room temperature. A fast reaction occurs, yielding a poorly soluble, light-yellow product. The analytical data are consistent with the proposed composition. Single crystals suitable for an X-ray diffraction study, obtained from cooling a saturated toluene solution, were isotypic with those previously measured. The quantum mechanical calculations also confirm the large stability of the product [Eu_2_(hfac)_6_(µ-Hq)_2_], **3**. Indeed, by considering the stability of the product as the difference between the energy of the product and the energies of the two reactants ([Eu(hfac)_3_] and Hq), a stabilization of over −50 kcal/mol is obtained. In particular, there is a very large enthalpic contribution, while, as expected, entropy disfavours the formation of a single moiety with respect to two separate reactants.

Considering a steric issue, it was realized that all chelate ligands previously used to prepare heterobimetallic dinuclear europium/aluminium compounds (meac, sal and naphthal) were able to form six-membered rings, while an 8-hydroxyquinolinato ligand only formed a five-membered ring. For this reason, the possibility of using the *N*-oxide 8-hydroxyquinoline, (HqNO) was evaluated, as the deprotonated form could afford a chelate six-membered ring ligand.

HqNO has already been reported in the literature [47], although its coordination chemistry is largely unexplored, with only a few reports of structurally characterized complexes; most of them involve the protonated neutral form [48,49,50] acting as a monodentate ligand through the *N*-oxide oxygen ligand, while the hydroxyl is involved in a hydrogen bond net. Only a single report detailing a deprotonated chelating coordination mode is present in the literature [51]. Our synthetic target [Al(qNO)_3_], **4**, appeared interesting, considering the extensive studies in the literature on the emission of substituted [Alq_3_] [52].

The reaction between [Al(O^t^Bu)_3_] and HqNO in a molar ratio of 1 to 3 has been carried out in toluene at the solvent refluxing temperature. The poorly soluble, yellow product [Al(qNO)_3_] was recovered at a high yield. The ATR-IR spectrum shows significant differences from the spectrum of the free ligand. Specifically, two strong ligand absorptions at 1276 and 888 cm^−1^ are absent in the spectrum of the product, showing that no free HqNO is present, and many differences in the form of wavenumber shifts or relative intensity changes can be noticed (Figures S2 and S3). Although relatively stable in the solid state, the infrared spectrum does not change for short air exposures; [Al(qNO)_3_] releases free HqNO in solution in the presence of traces of water. The ^1^H NMR spectrum of [Al(qNO)_3_] in an anhydrous CD_2_Cl_2_ solution showed a complex series of partially superimposed aromatic signals (Figures S4 and S5). In octahedral compounds with three nonsymmetric bidentate ligands, the *fac* isomer, having a C_3_ axis of symmetry, presents three magnetically equivalent ligands, while the three ligands of the *mer* isomer are magnetically nonequivalent and may present different sets of signals. Although a specific NMR study of the isomeric composition in solution was beyond the scope of this work, a comparison with the literature data reported for Alq_3_ [53,54] suggests the presence of both *fac* and *mer* isomers in the sample.

The reaction between [Ln(hfac)_3_] (Ln = Eu and Gd) and [Al(qNO)_3_] has been carried out in anhydrous DCE at room temperature. The reaction was monitored observing the dissolution of the initially sparingly soluble lanthanide precursor. The resulting yellow solution was evaporated at reduced pressure, yielding a yellow-orange residue that was recrystallized from toluene. The product is stable for short-term air exposures (IR in Figure S6) and is partially soluble in common organic solvents. 

**Figure 4:**
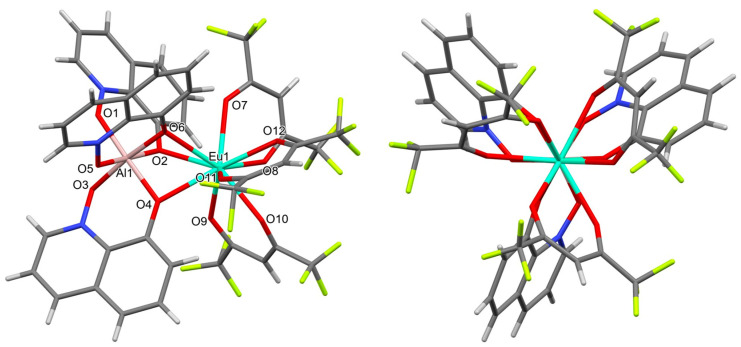
(**Left**) molecular structure of [Eu(hfac)_3_Al(qNO)_3_], **5**; (**Right**) projection along an approximated ternary axis. Lattice toluene has been omitted for clarity, (light green, europium; pink, aluminium; grey, carbon; red, oxygen; blue, nitrogen; yellow, fluorine).

Single crystals suitable for an X-ray diffraction study have been obtained by cooling at −20 °C in a saturated toluene solution for [Ln(hfac)_3_Al(qNO)_3_]: Ln = Eu (**5**) and Ln = Gd (**6**). They are isotypic. The molecular structure of **5** is reported in Figure 4.

The molecular structure shows a dinuclear compound, with the aluminium in a *fac* octahedral geometry and with three chelate qNO ligands sharing a trigonal face (O2, O4 and O6) with the tricapped trigonal prism of the nona-coordinated europium centre; three phenolic oxygen atoms are bridging the two metal centres. Although several attempts to crystallize [Al(qNO)_3_] failed, indirect information on its molecular structure has been acquired. It is possible to see an approximately ternary axis along the Eu–Al axis. (Figure 4). The calculated relative stabilities for the [Eu(hfac)_3_Alq_3_] and [Eu(hfac)_3_Al(qNO)_3_] can be estimated through DFT numerical simulations on the optimized structures of Al/Eu mononuclear complexes and the heterodinuclear ones. The Gibbs free energies of the formation reactions were taken as the difference between the Gibbs free energy of the heterodinuclear complex and the sum of those of the anhydrous [RE(hfac)_3_] fragment and [Al(qNO)_3_] or [Alq_3_] complexes. The calculated formation energies for [Eu(hfac)_3_Alq_3_] and [Eu(hfac)_3_Al(qNO)_3_] are −22.95 and −26.49 kcal/mol, where a more negative value indicates a more stable complex. The calculations suggest a higher relative stability for the [Eu(hfac)_3_Al(qNO)_3_] in agreement with the different experimental behaviour of the two complexes towards hydrolysis.

**Figure 5:**
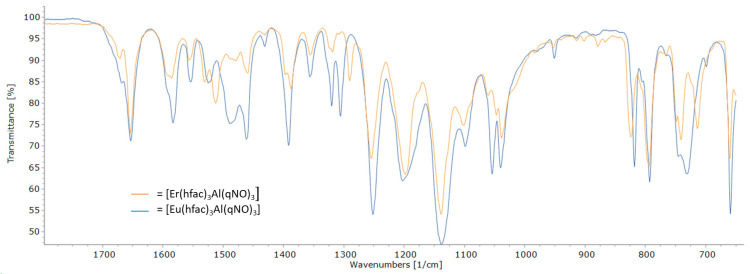
ATR-IR of [Er(hfac)_3_Al(qNO)_3_] (orange) and [Eu(hfac)_3_Al(qNO)_3_] (blue).

The possibility of obtaining an analogous compound for a late transition element such as erbium has been investigated. The anhydrous precursor [Er(hfac)_3_] has been suspended in toluene and treated with [Al(qNO)_3_] at room temperature. From the resulting solution, a single product has been recovered at a high yield. The analytical data are consistent with the composition [Er(hfac)_3_Al(qNO)_3_] (**7**). The infrared spectrum is similar to that of the europium analogue, although not completely superimposable (Figure 5). 

**Figure 6:**
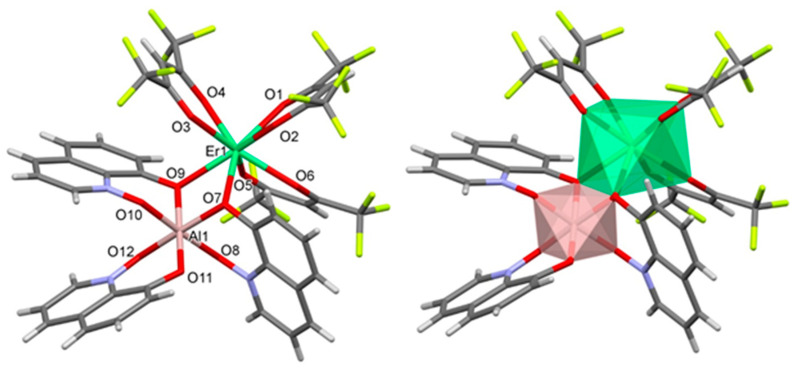
Molecular structure of [Er(hfac)_3_Al(qNO)_3_], **7**, with a polyhedral representation on the right, (green, erbium; pink, aluminium; grey, carbon; red, oxygen; blue, nitrogen; yellow, fluorine).

Single crystals suitable for an X-ray diffraction study have been obtained for the slow diffusion of pentane vapours in a DCE solution. The molecular structure of [Er(hfac)_3_Al(qNO)_3_] is shown in Figure 6. 

The heterometallic dinuclear compound shows the Er(hfac)_3_ fragment, with the metal completing its coordination sphere and receiving electron density from the Al(qNO)_3_ fragment. Different from the europium analogue, however, is that only two phenolic oxygen atoms are bridging the two metals, so that the two coordination polyhedra share an edge and not a face of the octahedron. It can be noticed that the Al(qNO)_3_ fragment is here in a *mer* disposition, and erbium has an almost ideal square antiprism geometry with a coordination number of 8. This difference can be rationalized considering the decrease in the ionic radius along the lanthanide series: 100.4 pm for erbium compared with 106.6 pm for europium [55].

Quantum mechanical calculations on these complexes can be useful to better rationalize the different geometrical arrangements (three or two phenolic bridging oxygen atoms) of the Eu/Gd and Er complexes, respectively. As described before, the relative stability of the structures can be evaluated as the difference between the Gibbs free energies of the heterometallic complexes [RE(hfac)_3_Al(qNO)_3_] with respect to the fragments [Al(qNO)_3_] and [RE(hfac)_3_], and the same strategy will also be used with complexes in which two oxygen atoms bridge the two fragments. To simplify calculations for rare-earth complexes, La is commonly chosen for its closed-shell electronic structure [32,56,57], with the idea that this replacement does not significantly alter the geometrical properties. For these heterometallic complexes, by considering [La(hfac)_3_Al(qNO)_3_], the structure with three bridging ligands is more stable than the one with two bridging ligands by about 4 kcal/mol (see Table 1). This result is in good agreement with the experimental molecular structures of europium and gadolinium but not with that of erbium. 

For this reason, the calculations were performed including explicitly the lanthanides Eu, Gd and Er. The energy differences obtained for Eu and Gd complexes, consistent with those calculated for La, show a preference for a molecular architecture presenting three bridging ligands (about 4 kcal/mol more stable, Table 1). This confirms that the replacement of Eu/Gd with La is a good approximation. The calculations for the Er complex show a larger stability for a molecular structure with only two bridging ligands, which is in agreement with the experimental data. Thus, the replacement of RE with La is valid only for larger lanthanide ions (Eu/Gd), while for smaller ones, it is necessary to explicitly include the RE.

In addition to the different ionic radii, it is also possible to quantify the Lewis acidity of the lanthanide site to better investigate the reason for these differences. The calculated condensed Fukui functions [58] reflect the reactivity of a site and its capability to accept (or lose) an electron, so that they identify the electrophilic (or nucleophilic) character of a molecule. For [RE(hfac)_3_], the acceptor condensed Fukui function $f_{k}^{+}$ provides information about the ability of the site to receive additional electron density in the molecular system: the higher the function, the higher the capability to accept an electron. Thus, it is expected that a site with a high acceptor condensed Fukui function $f_{k}^{+}$ will be a stronger Lewis acid. The calculated condensed Fukui functions $f_{k}^{+}$ for [RE(hfac)_3_] with RE = Eu, Gd and Er are 0.295, 0.433 and 0.055, respectively. These values suggest that the larger Eu and Gd ions are better Lewis acids (and electrophiles) with respect to the smaller Er ion. Previous spectroscopic studies carried out only in solution in the presence of water on [(Ln(tta)_3_)(Co(acac)_3_)] (Htta = 2-thenoyltrifluoroacetone) [59,60] reported a decrease in the stability of the Lewis acid to Lewis base interaction moving from lanthanum to lutetium, explained by the different kinds of structures for light and heavy lanthanides. They suggested that for early transition metals, a heterometallic dinuclear compound can form, while for late transition metals, their spectroscopic data could be explained by a hydrogen-bonding interaction between the Ln-coordinated water and Co(acac)_3_. The present finding clarifies that in anhydrous conditions, genuine heterometallic complexes [Ln(hfac)_3_Al(qNO)_3_] can also be obtained for late transition metals. A similar result has been highlighted elsewhere for [Ln(hfac)_3_Cu(acac)_2_)] complexes [61]. For [Ln(hfac)_3_Al(qNO)_3_] complexes, large early lanthanides significantly prefer the coordination number 9, with three donor atoms from three different qNO ligands lying on the same triangular face and bridging the two metal ions, while late transition metals possibly prefer the coordination number 8, with only two oxygen atoms bridging the two fragments. The calculated condensed Fukui function $f_{k}^{+}$ perfectly supports this sentence: the different electrophilicity can explain the different structure between the stronger Lewis acid (Eu and Gd) and the less electrophilic Er metal centre. 

Moreover, it is worth pointing out that the presence of only two oxygen atoms bridging the two fragments for the late transition metal Er does not mean a weaker bond energy. Indeed, the comparison between the relative stabilities (Gibbs free energies in Table 1) shows that the heterometallic Er complex is the most stable one. 

## 3. Materials and Methods

All manipulations were performed under a dinitrogen atmosphere using anhydrous solvents. [Alq_3_] [62,63] and HqNO [47] were synthesized according to the literature. Anhydrous [Ln(hfac)_3_] species (Ln^3+^ = Eu^3+^, Gd^3+^ and Er^3+^) were obtained by dehydration of the corresponding dihydrate complex [Ln(hfac)_3_(H_2_O)_2_] according to the procedure reported in the literature [64]. [Al(O^t^Bu)_3_] was purchased from Sigma Aldrich. ATR-IR spectra on solid samples were recorded with a Perkin–Elmer ‘‘Spectrum One’’ spectrometer, equipped with an ATR accessory. ^1^H NMR spectra were recorded with a Bruker “Avance DRX400” spectrometer. Chemical shifts were measured in ppm (δ) from TMS using residual solvent peaks. Elemental analyses (C, H and N) were performed using an Elementar “vario MICRO cube” instrument at Dipartimento di Chimica e Chimica Industriale, Università di Pisa. 

### 3.1. Reaction of [Eu(hfac)_3_] with [Alq_3_]

To a suspension of [Eu(hfac)_3_] (0.688 g; 0.89 mmol) in anhydrous toluene (70 mL), 0.410 g (0.89 mmol) of [Alq_3_] was added. After 3 days of stirring at room temperature, traces of an undissolved solid were filtered and discarded. From the filtrate, a yellow solid was recovered by removing the volatile phase in vacuo. (0.60 g; 54.4% yield for [Eu(hfac)_3_Alq_3_] (**1**). The elemental analysis calculated for [Eu(hfac)_3_Alq_3_] (C_42_H_21_N_3_AlEuF_18_O_9_): C: 41.0%, H: 1.7% and N: 3.4%; we found C: 41.4%, H: 2.0% and N: 3.5%. ATR-IR (range 1700–650 cm^−1^): 1650 (m), 1592 (w), 1556 (w), 1530 (w), 1503 (m), 1460 (w), 1421 (w), 1385 (w), 1320 (w), 1300 (w), 1253 (m), 1197 (m), 1132 (s), 1098 (m), 873 (w), 951 (w), 896 (w), 867 (w), 797 (m), 773 (w), 750 (w), 740 (w) and658 (m). In a recrystallization attempt of the product from hot heptane, in air, single crystals of two different morphologies and compositions were obtained: [Eu_2_(hfac)_6_Al_2_q_4_(OH)_2_] (**2**) and [Eu_2_(hfac)_6_(µ-Hq)_2_] (**3**). 

### 3.2. Synthesis of [Eu_2_(hfac)_6_(µ-Hq)_2_] (***3***)

To a suspension of [Eu(hfac)_3_] (0.275 g; 0.356 mmol) in toluene (40 mL), 8-hydroxyquinoline (0.052 g; 0.358 mmol) was added. The solid phase turned yellow. After 1 day of stirring, the yellow solid was filtered and dried (0.200 g; 61% yield for [Eu_2_(hfac)_6_(µ-Hq)_2_]). The elemental analysis calculated for [Eu_2_(hfac)_6_(µ-Hq)_2_] (Eu_2_C_48_H_20_O_14_N_2_F_36_): C: 31.4%, H: 1.1% and N: 1.5%; we found C: 31.0%, H: 1.3% and N: 1.4%. ATR-IR (range 1700–650 cm^−1^): 1651 (m), 1592 (w), 1556 (m), 1530 (m), 1505 (m), 1458 (w), 1421 (w), 1385 (w), 1350 (w), 1200 (w), 1253 (s), 1195 (s), 1135 (s), 1100 (s), 973 (w), 951 (w), 895 (w), 867 (w), 814 (m), 798 (s), 772 (m), 750 (w), 740 (w), 714 (w) and 658 (s). A further crystalline crop of the compound was recovered from cold toluene (−20 °C). Crystal data (**3**): triclinic, P $\bar{1}$, a = 12.002(**2**) Å, b = 12.830(**3**) Å, c = 13.117(**3**) Å, α = 107.53(**2**)°, β = 112.78(**2**)° and γ = 105.09(**1**)°.

### 3.3. Synthesis of [Al(qNO)_3_] (***4***)

A solution of HqNO (1.55 g; 9.63 mmol) in anhydrous toluene (80 mL) was treated with [Al(O^t^Bu)_3_] (0.79 g; 3.21 mmol) and heated at the reflux temperature for 4 h. The volatile phases were then removed in vacuo, leaving a yellow-brown residue that was suspended in toluene (20 mL) and filtered. (1.31 g; 80.4% yield for the title compound). It can be recrystallized through the slow diffusion of pentane vapours in a chloroform solution. Elemental analysis calculated for [Al(qNO)_3_] (C_27_H_18_N_3_AlO_6_): C: 63.9%, H: 3.6% and N: 8.3%; we found C: 63.6%, H: 3.3% and N: 8.2%. ATR-IR (range 1700–650 cm^−1^): 1587 (w), 1571 (m), 1513(w), 1460 (w), 1426 (w), 1389 (s), 1358 (w), 1321 (w), 1304 (w), 1217 (w), 1202 (w), 1167 (w), 1131 (w), 1087 (w), 1056 (w), 1039 (m), 826 (w), 813 (w), 789 (w), 759 (w) and 726 (w). ^1^H NMR (CD_2_Cl_2_): δ (ppm) 15.24 (s, 1H), 8.26 (d, 2H), 8.19 (dd, 18H), 8.04–7.97 (m, 38H), 7.84 (dt, 20H), 7.52 (t, 2H), 7.35–7.43 (m, 38H), 7.30–7.03 (m, 96H) and 6.72 (td, 38H). 

### 3.4. Synthesis of [Ln(hfac)_3_Al(qNO)_3_] (Ln = Eu, Gd and Er)

The synthesis of the europium derivative is described at length.

Eu (**5**): To a yellow suspension of [Al(qNO)_3_] (0.20 g; 0.39 mmol) in anhydrous 1,2-dichloroethane (DCE) (100 mL), [Eu(hfac)_3_] (0.30 g; 0.39 mmol) was added. The resulting solution was stirred overnight at room temperature and concentrated to dryness in vacuo. The yellow-orange residue was solubilized in toluene (10 mL) and precipitated with heptane. (0.34 g; 66% yield). Elemental analysis calculated for [Eu(hfac)_3_Al(qNO)_3_ ½ C_7_H_8_] (C_45_._5_H_25_N_3_AlF_18_EuO_12_) %: C: 41.2; H: 1.9; N: 3.2; we found C: 40.9, H: 1.7 and N: 3.2. ATR-IR (range 1700–650 cm^−1^): 1654 (d), 1583 (d), 1554 (d), 1525 (d), 1489 (d), 1461 (d), 1392 (d), 1356 (d), 1320 (d), 1306 (d), 1254 (d), 1202 (d), 1139 (d), 1094 (d), 1054 (m), 1038 (m), 1016 (m), 817 (d), 792 (f), 747 (d), 732 (d) and659 (d). The sample is stable in air for short-term exposures; no IR modification was noticed. Suitable crystals for X-ray diffraction were recovered from cold toluene (−20 °C). 

Gd (**6**): Starting from [Gd(hfac)_3_] (0.430 g; 0.55 mmol) and [Al(qNO)_3_] (0.278 g; 0.55 mmol) in toluene (25 mL), [Gd(hfac)_3_Al(qNO)_3_] was recovered (0.434 g; 59.2 yield). Elemental analysis calculated for [Gd(hfac)_3_Al(qNO)_3_ ½ C_7_H_8_] (C_45_._5_H_25_N_3_AlF_18_GdO_12_) %: C: 41.0; H: 1.9; N: 3.2; we found C: 40.7, H: 1.5 and N: 3.1. ATR-IR is almost completely superimposable with the europium analogue. Crystal data: monoclinic, P 2/c, a = 20.390(2) Å, b = 12.923(1) Å, c= 20.693(2) Å and β = 106.131(7)°.

Er (**7**): Starting from [Er(hfac)_3_] (0.46 g; 0.58 mmol) and [Al(qNO)_3_] (0.303 g; 0.60 mmol) in toluene (50 mL), [Er(hfac)_3_Al(qNO)_3_] was recovered (0.459 g; 61.0 yield). Elemental analysis calculated for [Er(hfac)_3_Al(qNO)_3_] (C_42_H_21_N_3_AlErF_18_O_12_): C: 38.9%, H: 1.6% and N: 3.2%; we found C: 38.6%, H: 1.5% and N: 3.1%. ATR-IR (range 1700–650 cm^−1^): 1653 (s), 1586 (m), 1556 (m), 1530 (m), 1513 (m), 1461 (m), 1431 (w), 1388 (m), 1356 (w), 1319 (w), 12090 (m), 1254 (s), 1198 (s), 1139 (vs), 1102 (m), 1039 (m), 1047 (m), 982 (w), 952 (w), 879 (w), 823 (s), 794 (s), 741 (s), 714 (s) and 660 (s). 

**X-ray diffraction studies.** Crystals were selected at room temperature (293 K), sealed in glass capillaries and analysed using a Bruker Smart Breeze CCD diffractometer equipped with Mo *Kα* radiation. The lattice parameters and some collection details are summarized in Table 2. After correction for Lorentz and polarization effects and for absorption, structures were solved with the ShelXT [65] program by intrinsic phasing and refined with the ShelXL [66] package using a least squares minimization. The crystal structure of **2** contains an asymmetric unit made up of two half molecules placed around two inversion centres. Two complete molecules with slightly different conformations are generated by the action of the symmetry operators. Some terminal CF_3_ groups are affected by a certain rotational disorder, which is reflected in rather elongated, calculated thermal ellipsoids. The disorder extent did not require a refinement on two different limit positions. The crystal structure of **3** contains an asymmetric unit consisting of half a molecule placed close to an inversion centre. The second half of the molecule is generated by the action of the $\bar{1}$ operator. The high disorder present in one hfac ligand required a refinement on two different limit positions, with total occupancy fixed to 1. The final values of the reliability factors obtained at the end of the refinement are reported in Table 2. The asymmetric unit of the **5** · 0.5 toluene crystal structure contains a molecule of toluene placed across a symmetry axis. The marked rotational disorder in one of the CF_3_ groups of the main compound required refinement on two different positions by fixing to 1 the total occupancy of the group. The high disorder also extends to the solvent molecule: introducing half a molecule of toluene into the model, an unacceptable geometry is obtained with refinement. For this reason, a whole toluene molecule, with a fixed geometry and occupancy of 0.5, was introduced into the model across the symmetry axis, affording a refinement convergence to the reliability factor reported in Table 2. The asymmetric unit of **7** corresponds to a single molecule. Also in this case, the rotational disorder of five of the six CF_3_ groups required a model with the disordered groups split into two different positions, with the total occupancy equal to 1. Table 2 reports the final reliability factors obtained in the refinement. Some control calculations were performed with the programs contained in the suite WINGX [67].

The crystallographic data for **2**, **3**, **5** and **7** reported in this paper have been deposited with the Cambridge Crystallographic Data Centre as supplementary publication no. CCDC 2298048-2298051. These data can be obtained free of charge from the Cambridge Crystallographic Data Centre via www.ccdc.cam.ac.uk/data_request/cif, (last access on 9 January 2024, CCDC: Cambridge, UK).

**Computational Details.** DFT calculations were carried out by using the Orca software (version 4.2.0) [68]. The GGA PBE functional [69,70], coupled to an all-electron, triple-ζ quality Ahlrichs basis set with one polarization function (def2-TZVP) [71] for all atoms, was employed to optimize the ground state molecular structures. Dispersion corrections were included by adopting Grimme’s DFT-D3 method [72]. The condensed Fukui functions, which reflect the reactivity of a site [58], were evaluated by carrying out a single-point calculation on the ground state geometry, first considering the neutral system and then considering it as singly charged. They were then calculated as the difference between the Mulliken atom charge in the two conditions. The condensed Fukui function $f_{k}^{+}$ on the atom k is defined as [73]: $f_{k}^{+}=q_{k}\left(N\right)-q_{k}\left(N+1\right)$, where $q_{k}$ is the Mulliken atom charge of atom k, calculated either in the neutral state with N electrons, or in the negatively charged molecule (N+1 electrons).

## 4. Conclusions

In this work, the synthetic protocol previously used to prepare heterobimetallic europium–aluminium complexes has been exploited, starting from [Eu(hfac)_3_] and [Alq_3_] as building blocks. Despite several attempts, the molecular compound [Eu(hfac)_3_Alq_3_] has not been crystallographically characterized, possibly being hindered by steric factors favouring a structural rearrangement promoted by a hydrolytic attack. Two different products have been structurally characterized from crystallization in the presence of water: the heterometallic [Eu_2_(hfac)_6_Al_2_q_4_(OH)_2_] and the homometallic, dinuclear europium derivative [Eu_2_(hfac)_6_(µ-Hq)_2_]. The latter can be directly prepared from [Eu(hfac)_3_] and 8-hydroxiquinoline at room temperature in toluene at a high yield, suggesting that this compound can be easily formed from the precursors, and that Hq is a better Lewis base than [Alq_3_] for the Lewis acid [Eu(hfac)_3_]. 

Using the six-membered chelating ring ligand qNO, an oxidized quinolinato ligand that has been rarely used in coordination chemistry, structural studies have been possible for [Ln(hfac)_3_Al(qNO)_3_] (Ln = Eu, Gd and Er). The phenolic oxygen atoms in [Al(qNO)_3_] are sufficiently basic to bridge the two metal centres and form the heterometallic compound. This result can be relevant in considering the necessary requisites of the chelate ligands on aluminium from a steric point of view. The synthetic protocol is utilizable also for late lanthanides, although the reduction in the ionic radius along the series affords a heterometallic compound with only two bridging oxygen atoms. These toluene-soluble heterometallic compounds are air stable in the solid state and present two close, different metal centres (about 3 Å). They are examples of a family of compounds that can be prepared along the lanthanide series. The quantum mechanics calculations correctly reproduce the different capabilities of the RE to bond with three or two oxygen atoms, and they justify this different behaviour in terms of the Lewis acidity by using calculated condensed Fukui functions. 

## Figures and Tables

**Table 1:** Calculated enthalpy (ΔH), entropy (TΔS at 298.15 K) and free Gibbs (ΔG) energies for a series of [RE(hfac)_3_Al(qNO)_3_] (RE= La, Er, Gd and Eu) complexes for two or three oxygen atoms bridging the two fragments. In the last column, the difference between Gibbs free energy for the bridging ligands (three oxygen atoms minus two oxygen atoms) is shown. All values are in kcal/mol.

	No. Bridging Ligands	Total Enthalpy/ΔH	Entropy/ −TΔS	Gibbs Free Energy/ΔG	ΔG Difference
[La(hfac)_3_Al(qNO)_3_]	2	−46.30	23.77	−22.53	
[La(hfac)_3_Al(qNO)_3_]	3	−52.67	26.18	−26.49	−3.96
[Eu(hfac)_3_Al(qNO)_3_]	2	−38.36	22.18	−16.18	
[Eu(hfac)_3_Al(qNO)_3_]	3	−42.46	22.43	−20.03	−3.85
[Gd(hfac)_3_Al(qNO)_3_]	2	−39.82	15.91	−23.91	
[Gd(hfac)_3_Al(qNO)_3_]	3	−42.94	15.10	−27.83	−3.92
[Er(hfac)_3_Al(qNO)_3_]	2	−53.02	22.08	−30.94	
[Er(hfac)_3_Al(qNO)_3_]	3	−50.04	21.01	−29.02	1.92

**Table 2:** Crystal data and refinement summaries for **2**, **3**, **5** and **7**.

**Identification Code**	**2**	**3**	**5** · 0.5 Toluene	**7**
CCDC number	2298051	2298049	2298048	2298050
Empirical formula	C_66_H_32_Al_2_Eu_2_F_36_N_4_O_18_	C_48_H_20_Eu_2_F_36_N_2_O_14_	C_45_._5_H_25_AlEuF_18_N_3_O_12_	C_42_H_21_AlErF_18_N_3_O_12_
Formula weight	2210.83	1836.58	1326.62	1295.86
Crystal system	Triclinic	Triclinic	Monoclinic	Triclinic
Space group	*P* $\bar{1}$	*P* $\bar{1}$	*P* 2/*c*	*P* $\bar{1}$
*a* (Å)	13.3724(5)	11.9995(7)	20.3737(6)	13.433(3)
*b* (Å)	17.3056(6)	12.8030(7)	12.9319(3)	13.522(2)
*c* (Å)	17.7157(7)	13.1061(7)	20.6857(6)	14.240(3)
*α* (°)	93.7570(10)	107.517(2)	-	91.094(6)
*β* (°)	92.5010(10)	112.709(2)	106.1290(10)	105.476(6)
*γ* (°)	98.2140(10)	105.019(2)	-	101.144(6)
Volume (Å^3^)	4043.1(3)	1601.95(16)	5235.6(2)	2439.0(8)
*Z*	2	1	4	2
*ρ*_calc_ (g cm^−1^)	1.816	1.904	1.683	1.765
*μ* (mm^−1^)	1.709	2.104	1.340	1.870
*F*(000)	2152	884	2612	1266
*θ* range (°)	1.19 to 26.50	3.26 to 27.48	2.59 to 25.99	2.67 to 26.39
Reflections collected	151373	54307	48953	85152
Data/restraints/parameters	16,701/0/1153	7314/210/505	10,231/43/741	9875/204/805
Goodness of fit on *F*^2^	1.019	1.132	1.054	1.125
Final *R*_1_ [*I* ≥ 2*σ*(*I*)]	0.0448	0.0380	0.0512	0.0510
Final *wR*_2_ [*I* ≥ 2*σ*(*I*)]	0.1191	0.0905	0.1290	0.1483
Final *R*_1_ [all data]	0.0684	0.0467	0.0791	0.0578
Final *wR*_2_ [all data]	0.1330	0.0995	0.1450	0.1559

Experiments were carried out at 294 K with Mo K*α* radiation.

## Data Availability

Data is contained within the article or Supplementary Material.

## Supplementary Materials

The following supporting information can be downloaded at: https://www.mdpi.com/article/10.3390/molecules29020451/s1. Figure S1: ATR-IR spectra (range 1700–650 cm^−1^) of [Eu(hfac)_3_Alq_3_] (black), [Alq_3_] (red) and [Eu(hfac)_3_(H_2_O)_2_] (blue). Figure S2: ATR-IR spectrum of 8-hydroxyquinoline *N*-oxide (HqNO). Figure S3: ATR-IR spectrum of [Al(qNO)_3_] (red) compared with [HqNO] (blue). Figure S4: ^1^H NMR of 8-hydroxyquinoline *N*-oxide (HqNO) in CDCl_3_. Figure S5: ^1^H NMR spectrum of [Al(qNO)_3_] in anhydrous CD_2_Cl_2_. Figure S6: ATR-IR spectra (1700–650 cm^−1^) of [Eu(hfac)_3_Al(qNO)_3_] (black), [Al(qNO)_3_] (red) and [HqNO] (blue).

## References

[1] Liu J., Xue J., Yang G.-P., Dang L.-L., Ma L.-F., Li D.-S., Wang Y.-Y. (2022). Recent advances of functional heterometallic-organic framework (HMOF) materials: Design strategies and applications. Coord. Chem. Rev..

[2] Bao S.-S., Qin M.-F., Zheng L.-M. (2020). Metal phosphonates incorporating metalloligands: Assembly, structures and properties. Chem. Commun..

[3] Abednatanzi S., Derakhshandeh P.G., Depauw H., Coudert F.-X., Vrielinck H., Van Der Voort P., Leus K. (2019). Mixed-metal metal–organic frameworks. Chem. Soc. Rev..

[4] Masoomi M.Y., Morsali A., Dhakshinamoorthy A., Garcia H. (2019). Mixed-Metal MOFs: Unique Opportunities in Metal–Organic Framework (MOF) Functionality and Design. Angew. Chem. Int. Ed..

[5] Liu K., Shi W., Cheng P. (2015). Toward heterometallic single-molecule magnets: Synthetic strategy, structures and properties of 3d–4f discrete complexes. Coord. Chem. Rev..

[6] Das M.C., Xiang S., Zhang Z., Chen B. (2011). Functional Mixed Metal–Organic Frameworks with Metalloligands. Angew. Chem. Int. Ed..

[7] Srivastava S., Gupta R. (2016). Metalloligands to material: Design strategies and network topologies. CrystEngComm.

[8] Kumar G., Gupta R. (2013). Molecularly designed architectures—The metalloligand way. Chem. Soc. Rev..

[9] Sørensen M.A., Weihe H., Vinum M.G., Mortensen J.S., Doerrer L.H., Bendix J. (2017). Imposing high-symmetry and tuneable geometry on lanthanide centres with chelating Pt and Pd metalloligands. Chem. Sci..

[10] Cruz C., Spodine E., Audebrand N., Venegas-Yazigi D., Paredes-Garciá V. (2018). Structural Versatility of 3*d*-Ce^III^ Heterometallic Coordination Polymers Using Co^II^ or Cu^II^. Cryst. Growth Des..

[11] Chandler B.D., Cramb D.T., Shimizu G.K.H. (2006). Microporous Metal−Organic Frameworks Formed in a Stepwise Manner from Luminescent Building Blocks. J. Am. Chem. Soc..

[12] Liu Y., Pan M., Yang Q.Y., Fu L., Li K., Wei S.C., Su C.Y. (2012). Dual-Emission from a Single-Phase Eu–Ag Metal–Organic Framework: An Alternative Way to Get White-Light Phosphor. Chem. Mater..

[13] Huang X.F., Ma J.X., Liu W.S. (2014). Lanthanide Metalloligand Strategy toward d–f Heterometallic Metal–Organic Frameworks: Magnetism and Symmetric-Dependent Luminescent Properties. Inorg. Chem..

[14] Ma J.X., Guo J., Wang H., Li B., Yang T., Chen B. (2017). Microporous Lanthanide Metal–Organic Framework Constructed from Lanthanide Metalloligand for Selective Separation of C_2_H_2_/CO_2_ and C_2_H_2_/CH_4_ at Room Temperature. Inorg. Chem..

[15] Qiu J.Z., Wang L.F., Chen Y.C., Zhang Z.M., Li Q.W., Tong M.L. (2016). Magnetocaloric Properties of Heterometallic 3d–Gd Complexes Based on the [Gd(oda)_3_]^3−^ Metalloligand. Chem.-A Eur. J..

[16] Visconti M., Maggini S., Ciani G., Mercandelli P., Del Secco B., Prodi L., Sgarzi M., Zaccheroni N., Carlucci L. (2019). New Lanthanide Metalloligands and Their Use for the Assembly of Ln–Ag Bimetallic Coordination Frameworks: Stepwise Modular Synthesis, Structural Characterization, and Optical Properties. Cryst. Growth Des..

[17] Merkens C., Englert U. (2012). Ordered bimetallic coordination networks featuring rare earth and silver cations. Dalton Trans..

[18] Yin J.C., Qin T.Z., Hu C., He G.M., Zhao B.W., Zhang C., Wang J. (2017). A copper(II)–gadolinium(III) heterometallic MOF: Synthesis, structure, and electrochemical property. Mater. Lett..

[19] Armelao L., Belli Dell’Amico D., Bellucci L., Bottaro G., Ciattini S., Labella L., Manfroni G., Marchetti F., Mattei C.A., Samaritani S. (2018). Homodinuclear Lanthanide Complexes with the Divergent Heterotopic 4,4′-Bipyridine N-Oxide (bipyMO) Ligand. Eur. J. Inorg. Chem..

[20] Bellucci L., Bottaro G., Labella L., Causin V., Marchetti F., Samaritani S., Belli Dell’Amico D., Armelao L. (2020). Composition−Thermometric Properties Correlations in Homodinuclear Eu^3+^ Luminescent Complexes. Inorg. Chem..

[21] Fioravanti L., Bellucci L., Armelao L., Bottaro G., Marchetti F., Pineider F., Poneti G., Samaritani S., Labella L. (2022). Stoichiometry Controlled Assembly of Lanthanide Molecular Complexes of the heteroditopic divergent ligand 4′-(4-pyridil)-2,2′:6′,2”-terpyridine *N*-oxide in hypodentate or bridging coordination modes. Structural, Magnetic and Photoluminescence studies. Inorg. Chem..

[22] Binnemans K. (2005). Rare-Earth Beta-Diketonates. Handb. Phys. Chem. Rare Earths.

[23] Lindoy L.F., Lip H.C., Louie H.W., Drew M.G.B., Hudson M.J. (1977). Interaction of lanthanide shift reagents with co-ordination complexes; direct observation of nuclear magnetic resonance signals for free and complexed tris(pentane-2,4-dionato)cobalt(III) at ambient temperature, and X-ray crystal and molecular structure. J. Chem. Soc. Chem. Commun..

[24] Øwre A., Vinum M., Kern M., van Slageren J., Bendix J., Perfetti M. (2018). Chiral, Heterometallic Lanthanide–Transition Metal Complexes by Design. Inorganics.

[25] Rogachev A.Y., Mironov A.V., Nemukhin A.V. (2007). Experimental and theoretical studies of the products of reaction between Ln(hfa)_3_ and Cu(acac)_2_ (Ln = La, Y; acac = acetylacetonate, hfa = hexafluoroacetylacetonate). J. Mol. Struct..

[26] Cosquer G., Pointillart F., Le Gal Y., Golhen S., Cador O., Ouahab L. (2011). Ferromagnetic versus Antiferromagnetic Exchange Interactions in Tetrathiafulvalene-Based 3d/4f Heterobimetallic Complexes. Chem. Eur. J..

[27] Kuz’mina N.P., Malkerova I.P., Alikhanyan A.S., Gleizes A.N. (2004). The use of 3d-metal complexes as ligands to prepare volatile 4f–3d heterobimetallic complexes. J. Alloys Compd..

[28] Jia R., Li H.-F., Chen P., Gao T., Sun W.-B., Li G.-M., Yan P.-F. (2016). Synthesis, structure, and tunable white light emission of heteronuclear Zn_2_Ln_2_ arrays using a zinc complex as ligand. CrystEngComm.

[29] Chen L., Breeze S.R., Rousseau R.J., Wang S., Thompson L.K. (1995). Polynuclear Copper-Lanthanide Complexes with Amino Alcohol Ligands. Syntheses, Structures, and Magnetic and Spectroscopic Studies of CuII(bdmmp)_2_(H_2_O), PrIIICuII(bdmmp)(bdmmpH)(µ-OH)(hfacac)_3_, [LaIIICuII(bdmmp)(bdmmpH)(µ-OH)(O_2_CCF_3_)_3_]_2_, and CuII_4_(bdmmp)_2_(µ_4_-O)(O_2_CCF_3_)_4_, (bdmmpH= 2,6-Bis[(dimethylamino)methyl]-4-methylphenol; hfacac = Hexafluoroacetylacetonato). Inorg. Chem..

[30] Armelao L., Belli Dell’Amico D., Bottaro G., Bellucci L., Labella L., Marchetti F., Mattei C.A., Mian F., Pineider F., Poneti G. (2018). 1D hetero-bimetallic regularly alternated 4f–3d coordination polymers based on N-oxide-4,4′-bipyridine (bipyMO) as a linker: Photoluminescence and magnetic properties. Dalton Trans..

[31] Bellucci L., Bottaro G., Labella L., Marchetti F., Samaritani S., Belli Dell’Amico D., Armelao L. (2021). 1D-Zigzag Eu^3+^/Tb^3+^ Coordination Chains as Luminescent Ratiometric Thermometers Endowed with Multicolor Emission. Materials.

[32] Bellucci L., Babetto L., Bottaro G., Carlotto S., Labella L., Gallo E., Marchetti F., Samaritani S., Armelao L. (2023). Competing excitation paths in Luminescent Heterobimetallic Ln-Al complexes: Unravelling interactions via experimental and theoretical investigations. iScience.

[33] Fischbach A., Anwander R. (2006). Rare-earth metals and aluminum getting close in Ziegler-type organometallics. Adv. Polym. Sci..

[34] Xu H.B., Deng J.G., Kang B. (2013). Designed synthesis and photophysical properties of multifunctional hybrid lanthanide complexes. RSC Adv..

[35] Westin G., Ekstrand Å., Zangellini E., Börjesson L. (2000). Preparation and optical studies of Er-doped Al-Si-Ti oxide glasses using the ErAl_3_(OPr^i^)_12_ isolated Er-ion precursor. J. Phys. Chem. Solids.

[36] Khan M.B., Salah ·N., Khan Z.H. (2022). Functional enhancement in Alq3 via metal doping and nanoscale synthesis: A review. Appl. Nanosci..

[37] Brinkmann M., Gadret G., Muccini M., Taliani C., Masciocchi N., Sironi A. (2000). Correlation between Molecular Packing and Optical Properties in Different Crystalline Polymorphs and Amorphous Thin Films of *mer*-Tris(8-hydroxyquinoline)aluminum(III). J. Am. Chem. Soc..

[38] Tang C.W., Vanslyke S.A. (1987). Organic electroluminescent diodes. Appl. Phys. Lett..

[39] Miyamae T., Ito E., Noguchi Y., Ishii H. (2011). Characterization of the Interactions between Alq_3_ Thin Films and Al Probed by Two-Color Sum-Frequency Generation Spectroscopy. J. Phys. Chem. C.

[40] Friend R.H., Gymer R.W., Holmes A.B., Burroughes J.H., Marks R.N., Taliani C., Bradley D.D.C., Dos Santos D.A., BreÂdas J.L., Lo Ègdlund M. (1999). Electroluminescence in Conjugated Polymers. Nature.

[41] Hu J.S., Ji H.X., Cao A.M., Huang Z.X., Zhang Y., Wan L.J., Xia A.D., Yu D.P., Meng X.M., Lee S.T. (2007). Facile solution synthesis of hexagonal Alq_3_ nanorods and their field emission properties. Chem. Commun..

[42] Aziz H., Popovic Z., Xie S., Hor A.M., Hu N.X., Tripp C., Xu G. (1998). Humidity-induced crystallization of tris (8-hydroxyquinoline) aluminum layers in organic light-emitting devices. Appl. Phys. Lett..

[43] Utz M., Chen C., Morton M., Papadimitrakopoulos F. (2003). Ligand Exchange Dynamics in Aluminum Tris-(Quinoline-8-olate):  A Solution State NMR Study. J. Am. Chem. Soc..

[44] Xu H.B., Chen X.M., Zhang Q.S., Zhang L.Y., Chen Z.N. (2009). Fluoride-enhanced lanthanide luminescence and white-light emitting in multifunctional Al_3_Ln_2_ (Ln = Nd, Eu, Yb) heteropentanuclear complexes. Chem. Commun..

[45] Xu H.B., Zhang L.Y., Huang X., Li B., Chen Z.N. (2010). Structures and Photophysical Properties of Homo- and Heteronuclear Lanthanide(III) Complexes with Bridging 2-Methyl-8-hydroxylquinoline (HMq) in the μ-Phenol Mode. Cryst. Growth Des..

[46] Tsuboi T., Torii Y. (2010). Selective Synthesis of Facial and Meridianal Isomers of Alq_3_. Mol. Cryst. Liq. Cryst..

[47] Shrader W.D., Celebuski J., Kline S.J., Johnson D. (1988). Synthesis of a novel hexadentate chelating agent based on 8-hydroxyquinoline. Tetrahedron Lett..

[48] Zhang S.G., Xu H.Y., Shi J.M. (2007). The 2:1 Adduct of 8-Hydroxyquinoline N-Oxide and Diaquabis(8-Hydroxy-Quinoline N-Oxide)Dithiocyanato-Cobalt(II). Acta Crystallogr. Sect. E Struct. Rep. Online.

[49] Shi J.M., Meng X.Z., Sun Y.M., Xu H.Y., Shi W., Cheng P., Liu L.D. (2009). Magnetic Study of a One-Dimensional Mn(II) Coordination Polymer Dealing with π-π Stacking. J. Mol. Struct..

[50] Gu L.Q., Li J.M. (2010). Catena-Poly[[[Diaquabis(8-Hydroxy-Quinoline N-Oxide-ΚO 1)Cobalt(II)] μ-2,5-Dimethyl-Pyrazine 1,4-Dioxide- Κ2 O 1:O 4] Bis-(Perchlorate)]. Acta Crystallogr. Sect. E Struct. Rep. Online.

[51] Yang Y., Zhou Z., Wei Z.-Z., Qin Q.-P., Yang L., Liang H. (2021). High anticancer activity and apoptosis- and autophagy-inducing properties of novel lanthanide(III) complexes bearing 8-hydroxyquinoline-N-oxide and 1,10-phenanthroline. Dalton Trans..

[52] Montes V.A., Li G., Pohl R., Shinar J., Anzenbacher P. (2004). Effective Color Tuning in Organic Light-Emitting Diodes Based on Aluminum Tris(5-Aryl-8-Hydroxyquinoline) Complexes. Adv. Mater..

[53] Rajeswaran M., Blanton T.N., Tang C.W., Lenhart W.C., Switalski S.C., Giesen D.J., Antalek B.J., Pawlik T.D., Kondakov D.Y., Zumbulyadis N. (2009). Structural, thermal, and spectral characterization of the different crystalline forms of Alq3, tris(quinolin-8-olato)aluminum(III), an electroluminescent material in OLED technology. Polyhedron.

[54] Lima C.F.R.A.C., Taveira R.J.S., Costa J.C.S., Fernandes A.M., Melo A., Silva A.M.S., Santos L.M.N.B.F. (2016). Understanding M–ligand bonding and mer-/facisomerism in tris(8-hydroxyquinolinate) metallic complexes. Phys. Chem. Chem. Phys..

[55] Shannon R.D. (1976). Revised Effective Ionic Radii and Systematic Studies of Interatomic Distances in Halides and Chalcogenides. Acta Crystallogr. Sect..

[56] Carlotto A., Babetto L., Carlotto S., Miozzi M., Seraglia R., Casarin M., Bottaro G., Rancan M., Armelao L. (2020). Luminescent Thermometers: From a Library of Europium(III) β-Diketonates to a General Model for Predicting the Thermometric Behaviour of Europium-Based Coordination Systems. ChemPhotoChem.

[57] Rancan M., Tessarolo J., Carlotto A., Carlotto S., Rando M., Barchi L., Bolognesi E., Seraglia R., Bottaro G., Casarin M. (2022). Adaptive helicity and chiral recognition in bright europium quadruple-stranded helicates induced by host-guest interaction. Cell Rep. Phys. Sci..

[58] Parr R.G., Yang W. (1984). Density Functional Approach to the Frontier-Electron Theory of Chemical Reactivity. J. Am. Chem. Soc..

[59] Kameta N., Imura H., Ohashi K., Aoyama T. (1999). Equilibrium and Spectroscopic Studies on the Complexation of Tris(1-(2-Thienyl)-4,4,4-Trifluoro-1,3-Butanedionato)Lanthanoids(III) with Tris(2,4-Pentanedionato)Cobalt(III) as Complex Ligand. Inorg. Chem. Commun..

[60] Kameta N., Imura H., Ohashi K., Aoyama T. (2002). Stability Constants of Inner- and Outer-Sphere Complexes of Hydrated Tris(1-(2-Thienyl)-4,4,4-Trifluoro-1,3-Butanedionato) Lanthanide(III) with Tris(2,4-Pentanedionato) Cobalt(III). Polyhedron.

[61] Bellucci L., Giordano L., Carlotto S., Poneti G., Rancan M., Samaritani S., Armelao L., Labella L. (2023). Heterometallic [Ln(hfac)_3_Cu(acac)_2_] complexes with late 4*f* ions. Inorg. Chim. Acta.

[62] Li H., Zhang F., Wang Y., Zheng D. (2003). Synthesis and characterization of tris-(8-hydroxyquinoline)aluminum. Mater. Sci. Eng. B.

[63] Katakura R., Koide Y. (2006). Configuration-Specific Synthesis of the Facial and Meridional Isomers of Tris(8-hydroxyquinolinate)aluminum (Alq_3_). Inorg. Chem..

[64] Armelao L., Belli Dell’Amico D., Bellucci L., Bottaro G., Labella L., Marchetti F., Samaritani S. (2016). A convenient synthesis of highly luminescent lanthanide 1D-zigzag coordination chains based only on 4,4′-bipyridine as connector. Polyhedron.

[65] Sheldrick G.M. (2015). SHELXT—Integrated Space-Group and Crystal-Structure Determination. Acta Crystallogr. Sect. A Found. Crystallogr..

[66] Sheldrick G.M. (2015). Crystal Structure Refinement with SHELXL. Acta Crystallogr. Sect. C Struct. Chem..

[67] Farrugia L.J. (2012). WinGX and ORTEP for Windows: An Update. J. Appl. Crystallogr..

[68] Neese F. (2018). Software update: The ORCA program system, version 4.0. Wiley Interdiscip. Rev. Comput. Mol. Sci..

[69] Perdew J.P., Wang Y. (1992). Accurate and simple analytic representation of the electron-gas correlation energy. Phys. Rev. B.

[70] Perdew J.P., Burke K., Ernzerhof M. (1996). Generalized Gradient Approximation Made Simple. Phys. Rev. Lett..

[71] Weigend F., Ahlrichs R. (2005). Balanced basis sets of split valence, triple zeta valence and quadruple zeta valence quality for H to Rn: Design and assessment of accuracy. Phys. Chem. Chem. Phys..

[72] Grimme S., Antony J., Ehrlich S., Krieg H. (2010). A consistent and accurate *ab initio* parametrization of density functional dispersion correction (DFT-D) for the 94 elements H-Pu. J. Chem. Phys..

[73] Yang W., Mortier W.J. (1986). The Use of Global and Local Molecular Parameters for the Analysis of the Gas-Phase Basicity of Amines. J. Am. Chem. Soc..

