# Supplementary material for: Aluminium 8-Hydroxyquinolinate N-Oxide as a Precursor to Heterometallic Aluminium–Lanthanide Complexes
Source: Molecules. 2024 Jan 17;29(2):451. doi: 10.3390/molecules29020451 (PMC10821320; doi:10.3390/molecules29020451)

checkCIF/PLATON report

Structure factors have been supplied for datablock(s) shelx

THIS REPORT IS FOR GUIDANCE ONLY. IF USED AS PART OF A REVIEW PROCEDURE FOR PUBLICATION, IT SHOULD NOT REPLACE THE EXPERTISE OF AN EXPERIENCED CRYSTALLOGRAPHIC REFEREE.

No syntax errors found. CIF dictionary Interpreting this report

Datablock: shelx

Bond precision:	C-C = 0.0139 A		Wavelength=0.71073
Cell:	a=20.3737(6)	b=12.9319(3)	c=20.6857(6)
	alpha=90	beta=106.129(1)	gamma=90
Temperature:	296 K		

	Calculated	Reported
Volume	5235.6(2)	5235.6(2)
Space group	P 2/c	P 2/c
Hall group	-P 2yc	-P 2yc
Moiety formula	2(C42 H21 Al Eu F18 N3 O12), C7 H8	C42 H21 Al Eu F18 N3 O12, 0.5(C7 H8)
Sum formula	C91 H50 Al2 Eu2 F36 N6 O24	C45.50 H25 Al Eu F18 N3 O12
Mr	2653.27	1326.62
Dx,g cm-3	1.683	1.683
Z	2	4
Mu (mm-1)	1.340	1.340
F000	2612.0	2612.0
F000'	2613.67	
h,k,lmax	25,15,25	25,15,25
Nref	10293	10231
Tmin,Tmax	0.709,0.913	0.568,0.745
Tmin'	0.690	

Correction method= # Reported T Limits: Tmin=0.568 Tmax=0.745
AbsCorr = MULTI-SCAN

Data completeness= 0.994 Theta(max)= 25.993

R(reflections)= 0.0512(7219) wR2(reflections)= 0.1450(10231)

S = 1.054 Npar= 741

The following ALERTS were generated. Each ALERT has the format
test-name_ALERT_alert-type_alert-level.
Click on the hyperlinks for more details of the test.

Alert level B

```
PLAT234_ALERT_4_B Large Hirshfeld Difference C44      --C45      .       0.26 Ang.
PLAT910_ALERT_3_B Missing # of FCF Reflection(s) Below Theta(Min).         13 Note
                0  1  0,   1  0  0,   1  1  0,   2  0  0,  -2  1  1,  -1  1  1,
                0  1  1,   1  1  1,  -2  0  2,  -1  0  2,  -1  1  2,   0  0  2,
                1  0  2,
```

Alert level C

```
PLAT042_ALERT_1_C Calc. and Reported MoietyFormula Strings  Differ     Please Check
              Calc: 2(C42 H21 Al Eu F18 N3 O12), C7 H8
              Rep.: C42 H21 Al Eu F18 N3 O12, 0.5(C7 H8)
PLAT213_ALERT_2_C Atom F4             has ADP max/min Ratio .....         3.3 prolat
PLAT220_ALERT_2_C NonSolvent   Resd 1  C   Ueq(max)/Ueq(min) Range        3.9 Ratio
PLAT230_ALERT_2_C Hirshfeld Test Diff for    F18      --C42      .        5.3 s.u.
PLAT230_ALERT_2_C Hirshfeld Test Diff for    O10      --C36      .        5.3 s.u.
PLAT230_ALERT_2_C Hirshfeld Test Diff for    O12      --C41      .        5.4 s.u.
PLAT234_ALERT_4_C Large Hirshfeld Difference F4       --C32      .       0.19 Ang.
PLAT234_ALERT_4_C Large Hirshfeld Difference F6       --C32      .       0.18 Ang.
PLAT234_ALERT_4_C Large Hirshfeld Difference F7A      --C33      .       0.22 Ang.
PLAT234_ALERT_4_C Large Hirshfeld Difference F8A      --C33      .       0.24 Ang.
PLAT234_ALERT_4_C Large Hirshfeld Difference F11      --C37      .       0.22 Ang.
PLAT234_ALERT_4_C Large Hirshfeld Difference F13      --C38      .       0.22 Ang.
PLAT234_ALERT_4_C Large Hirshfeld Difference F14      --C38      .       0.24 Ang.
PLAT234_ALERT_4_C Large Hirshfeld Difference F15      --C38      .       0.19 Ang.
PLAT234_ALERT_4_C Large Hirshfeld Difference F16      --C42      .       0.18 Ang.
PLAT234_ALERT_4_C Large Hirshfeld Difference O8       --C31      .       0.16 Ang.
PLAT234_ALERT_4_C Large Hirshfeld Difference O11      --C39      .       0.16 Ang.
PLAT234_ALERT_4_C Large Hirshfeld Difference N1       --C9       .       0.16 Ang.
PLAT234_ALERT_4_C Large Hirshfeld Difference C34      --C35      .       0.16 Ang.
PLAT241_ALERT_2_C High   'MainMol' Ueq as Compared to Neighbors of         O1 Check
PLAT241_ALERT_2_C High   'MainMol' Ueq as Compared to Neighbors of         C2 Check
PLAT241_ALERT_2_C High   'MainMol' Ueq as Compared to Neighbors of         C3 Check
PLAT241_ALERT_2_C High   'MainMol' Ueq as Compared to Neighbors of         C5 Check
PLAT241_ALERT_2_C High   'MainMol' Ueq as Compared to Neighbors of        C20 Check
PLAT241_ALERT_2_C High   'MainMol' Ueq as Compared to Neighbors of        C21 Check
PLAT241_ALERT_2_C High   'MainMol' Ueq as Compared to Neighbors of        C23 Check
PLAT242_ALERT_2_C Low    'MainMol' Ueq as Compared to Neighbors of        Eu1 Check
PLAT242_ALERT_2_C Low    'MainMol' Ueq as Compared to Neighbors of         C4 Check
PLAT242_ALERT_2_C Low    'MainMol' Ueq as Compared to Neighbors of        C19 Check
PLAT242_ALERT_2_C Low    'MainMol' Ueq as Compared to Neighbors of        C27 Check
PLAT242_ALERT_2_C Low    'MainMol' Ueq as Compared to Neighbors of        C31 Check
PLAT242_ALERT_2_C Low    'MainMol' Ueq as Compared to Neighbors of        C36 Check
PLAT242_ALERT_2_C Low    'MainMol' Ueq as Compared to Neighbors of        C39 Check
PLAT242_ALERT_2_C Low    'MainMol' Ueq as Compared to Neighbors of        C41 Check
PLAT244_ALERT_4_C Low    'Solvent' Ueq as Compared to Neighbors of        C46 Check
PLAT260_ALERT_2_C Large Average Ueq of Residue Including       Eu1      0.150 Check
PLAT260_ALERT_2_C Large Average Ueq of Residue Including       C43      0.212 Check
PLAT342_ALERT_3_C Low Bond Precision on  C-C Bonds ..............     0.01389 Ang.
PLAT601_ALERT_2_C Unit Cell Contains Solvent Accessible VOIDS of .         40 Ang**3
PLAT721_ALERT_1_C Bond    Calc     0.97000, Rep     0.99(4) Dev...       0.02 Ang.
```

```
              C47      -H47E            1_555   2_756 ........     #  132 Check
PLAT911_ALERT_3_C Missing FCF Refl Between Thmin & STh/L=    0.600         44 Report
                0  3  0,   2  1  0,   2  2  0,   3  0  0,   4  0  0,   5  1  0,
                6 14  0,  -5  1  1,  -4  2  1,  -3  1  1,  -1  2  1,   1  2  1,
                3  1  1,   3  2  1,   5  2  1,   5 14  1,  -5  0  2,  -3  0  2,
               -2  1  2,  -2  3  2,   1  1  2,   1  3  2,   2  0  2,   2  2  2,
                3  0  2,   4  2  2,  -4  1  3,  -4  2  3,  -3  2  3,  -1  1  3,
                0  1  3,   0  2  3,   1  1  3,   1  2  3,   2  2  3,   3  1  3,
                4  1  3,  -3  0  4,  -3  1  4,   0  0  4,   2  0  4,  -4  4  5,
               -2  1  5,  -1  0  6,
PLAT913_ALERT_3_C Missing # of Very Strong Reflections in FCF ....         15 Note
                0  3  0,   2  0  0,  -4  2  1,  -1  1  1,   1  1  1,  -5  0  2,
                0  0  2,   3  0  2,   4  2  2,  -4  1  3,  -3  2  3,   3  1  3,
                0  0  4,  -4  4  5,  -2  1  5,
```

Alert level G

```
FORMU01_ALERT_1_G  There is a discrepancy between the atom counts in the
            _chemical_formula_sum and _chemical_formula_moiety. This is
            usually due to the moiety formula being in the wrong format.
            Atom count from _chemical_formula_sum:   C45.5 H25 Al1 Eu1 F18 N3 O12
            Atom count from _chemical_formula_moiety:C45.5 H25 Al1 Eu1 F18 N3012
FORMU01_ALERT_2_G  There is a discrepancy between the atom counts in the
            _chemical_formula_sum and the formula from the _atom_site* data.
            Atom count from _chemical_formula_sum:C45.5 H25 Al1 Eu1 F18 N3 O12
            Atom count from the _atom_site data:  C46.5 H25 Al1 Eu1 F18 N3 O12
CELLZ01_ALERT_1_G Difference between formula and atom_site contents detected.
CELLZ01_ALERT_1_G ALERT: Large difference may be due to a
            symmetry error - see SYMMG tests
           From the CIF: _cell_formula_units_Z    4
           From the CIF: _chemical_formula_sum  C45.50 H25 Al Eu F18 N3 O12
           TEST: Compare cell contents of formula and atom_site data

           atom    Z*formula  cif sites diff
           C        182.00    186.00   -4.00
           H        100.00    100.00    0.00
           Al         4.00      4.00    0.00
           Eu         4.00      4.00    0.00
           F         72.00     72.00    0.00
           N         12.00     12.00    0.00
           O         48.00     48.00    0.00
PLAT002_ALERT_2_G Number of Distance or Angle Restraints on AtSite          1 Note
PLAT003_ALERT_2_G Number of Uiso or Uij Restrained non-H Atoms ...         10 Report
PLAT045_ALERT_1_G Calculated and Reported Z Differ by a Factor ...      0.500 Check
PLAT083_ALERT_2_G SHELXL Second Parameter in WGHT  Unusually Large       6.98 Why ?
PLAT172_ALERT_4_G The CIF-Embedded .res File Contains DFIX Records          1 Report
PLAT178_ALERT_4_G The CIF-Embedded .res File Contains SIMU Records          3 Report
PLAT186_ALERT_4_G The CIF-Embedded .res File Contains ISOR Records          1 Report
PLAT188_ALERT_3_G A Non-default SIMU Restraint Value has been used     0.0100 Report
PLAT188_ALERT_3_G A Non-default SIMU Restraint Value has been used     0.0100 Report
PLAT188_ALERT_3_G A Non-default SIMU Restraint Value has been used     0.0200 Report
PLAT242_ALERT_2_G Low    'MainMol' Ueq as Compared to Neighbors of        C28 Check
PLAT242_ALERT_2_G Low    'MainMol' Ueq as Compared to Neighbors of        C32 Check
PLAT242_ALERT_2_G Low    'MainMol' Ueq as Compared to Neighbors of        C33 Check
PLAT242_ALERT_2_G Low    'MainMol' Ueq as Compared to Neighbors of        C37 Check
PLAT242_ALERT_2_G Low    'MainMol' Ueq as Compared to Neighbors of        C42 Check
PLAT300_ALERT_4_G Atom Site Occupancy of H47A       Constrained at       0.25 Check
```

```
PLAT300_ALERT_4_G Atom Site Occupancy of H47B       Constrained at       0.25 Check
PLAT300_ALERT_4_G Atom Site Occupancy of H47C       Constrained at       0.25 Check
PLAT300_ALERT_4_G Atom Site Occupancy of H47D       Constrained at       0.25 Check
PLAT300_ALERT_4_G Atom Site Occupancy of H47E       Constrained at       0.25 Check
PLAT300_ALERT_4_G Atom Site Occupancy of H47F       Constrained at       0.25 Check
PLAT301_ALERT_3_G Main Residue  Disorder ..............(Resd    1)         4% Note
PLAT367_ALERT_2_G Long?  C(sp?)-C(sp?) Bond  C46      - C47      .       1.51 Ang.
PLAT434_ALERT_2_G Short Inter HL..HL Contact F13      ..F13      .       2.70 Ang.
                                            1-x,y,1/2-z  =      2_655 Check
PLAT721_ALERT_1_G Bond    Calc     0.97000, Rep     0.96000 Dev...       0.01 Ang.
              C47      -H47A           1_555   1_555 ........     #  124 Check
PLAT721_ALERT_1_G Bond    Calc     0.97000, Rep     0.96000 Dev...       0.01 Ang.
              C47      -H47E           1_555   1_555 ........     #  128 Check
PLAT722_ALERT_1_G Angle   Calc      108.00, Rep      109.50 Dev...       1.50 Degree
              H47A    -C47     -H47C       1_555  1_555  1_555     #  260 Check
PLAT722_ALERT_1_G Angle   Calc      111.00, Rep      109.50 Dev...       1.50 Degree
              H47D    -C47     -H47F       1_555  1_555  1_555     #  263 Check
PLAT722_ALERT_1_G Angle   Calc      108.00, Rep      109.50 Dev...       1.50 Degree
              H47E    -C47     -H47F       1_555  1_555  1_555     #  264 Check
PLAT860_ALERT_3_G Number of Least-Squares Restraints .............         43 Note
PLAT883_ALERT_1_G No Info/Value for _atom_sites_solution_primary .     Please Do !
PLAT899_ALERT_4_G SHELXL2018 is Deprecated and Succeeded by SHELXL     2019/3 Note
PLAT912_ALERT_4_G Missing # of FCF Reflections Above STh/L=  0.600          5 Note
PLAT933_ALERT_2_G Number of HKL-OMIT Records in Embedded .res File         17 Note
                2  0  0,   0  0  2,  -1  1  1,   1  1  1,  -3  0  2,  -1  0  2,
                0  1  3,   3  1  1,   4  0  0,   0  1  1,  -2  0  2,  -1  1  3,
               -2  1  1,   1  0  0,   0  1  0,   1  1  0,  -1  2  1,
PLAT941_ALERT_3_G Average HKL Measurement Multiplicity ...........        4.8 Low
PLAT965_ALERT_2_G The SHELXL WEIGHT Optimisation has not Converged     Please Check
PLAT969_ALERT_5_G The 'Henn et al.' R-Factor-gap value ...........       4.96 Note
              Predicted wR2: Based on SigI**2  2.92 or SHELX Weight 14.28
PLAT978_ALERT_2_G Number C-C Bonds with Positive Residual Density.          0 Info
```

```
   0 ALERT level A = Most likely a serious problem - resolve or explain
   2 ALERT level B = A potentially serious problem, consider carefully
  42 ALERT level C = Check. Ensure it is not caused by an omission or oversight
  42 ALERT level G = General information/check it is not something unexpected

  12 ALERT type 1 CIF construction/syntax error, inconsistent or missing data
  37 ALERT type 2 Indicator that the structure model may be wrong or deficient
  10 ALERT type 3 Indicator that the structure quality may be low
  26 ALERT type 4 Improvement, methodology, query or suggestion
   1 ALERT type 5 Informative message, check
```

It is advisable to attempt to resolve as many as possible of the alerts in all categories. Often the minor alerts point to easily fixed oversights, errors and omissions in your CIF or refinement strategy, so attention to these fine details can be worthwhile. In order to resolve some of the more serious problems it may be necessary to carry out additional measurements or structure refinements. However, the purpose of your study may justify the reported deviations and the more serious of these should normally be commented upon in the discussion or experimental section of a paper or in the "special_details" fields of the CIF. checkCIF was carefully designed to identify outliers and unusual parameters, but every test has its limitations and alerts that are not important in a particular case may appear. Conversely, the absence of alerts does not guarantee there are no aspects of the results needing attention. It is up to the individual to critically assess their own results and, if necessary, seek expert advice.

Publication of your CIF in IUCr journals

A basic structural check has been run on your CIF. These basic checks will be run on all CIFs submitted for publication in IUCr journals (*Acta Crystallographica*, *Journal of Applied Crystallography*, *Journal of Synchrotron Radiation*); however, if you intend to submit to *Acta Crystallographica Section C* or *E* or *IUCrData*, you should make sure that full publication checks are run on the final version of your CIF prior to submission.

Publication of your CIF in other journals

Please refer to the *Notes for Authors* of the relevant journal for any special instructions relating to CIF submission.

PLATON version of 13/12/2023; check.def file version of 13/12/2023

Datablock shelx - ellipsoid plot

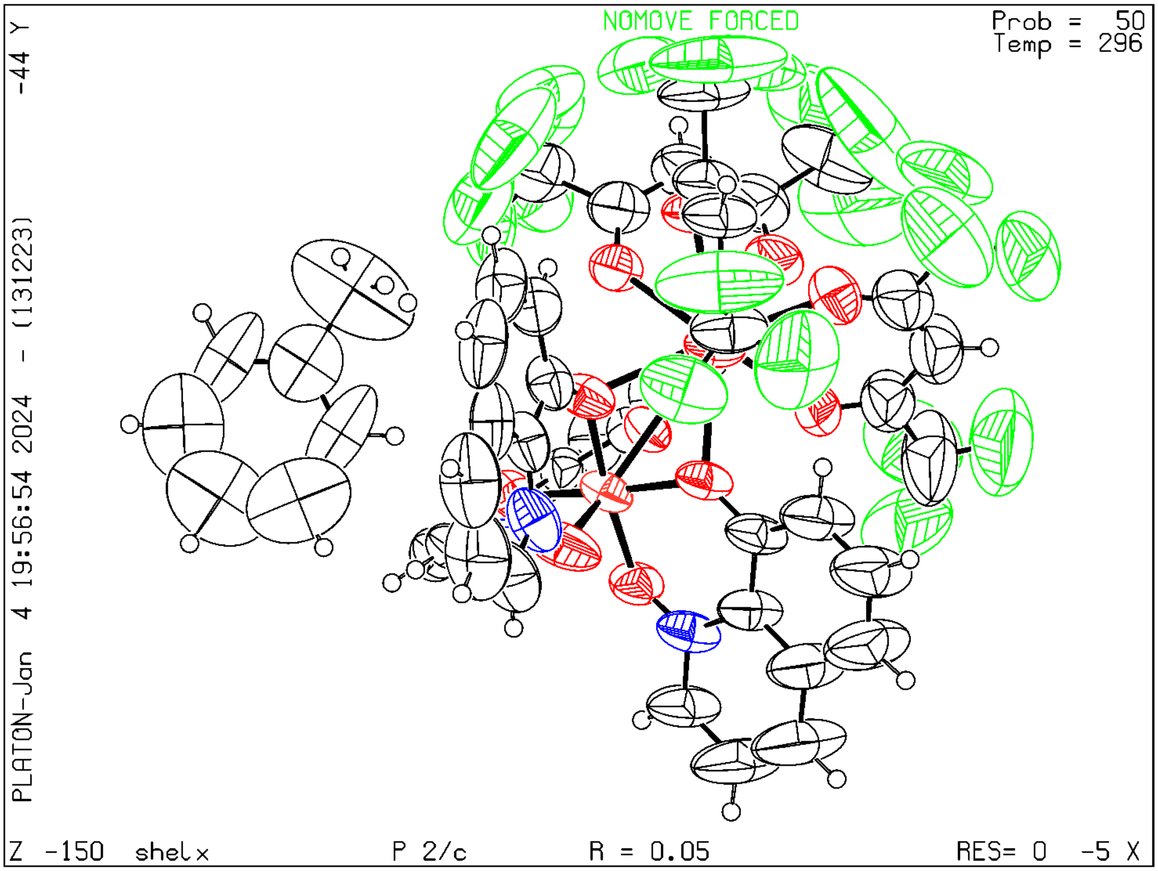

Supplement: Supplementary file 1 [file molecules-29-00451-s001.zip › checkcif_Compn_5.pdf]
