# Supplementary material for: Aluminium 8-Hydroxyquinolinate N-Oxide as a Precursor to Heterometallic Aluminium–Lanthanide Complexes
Source: Molecules. 2024 Jan 17;29(2):451. doi: 10.3390/molecules29020451 (PMC10821320; doi:10.3390/molecules29020451)
Supplement: Supplementary file 1 [file molecules-29-00451-s001.zip › molecules-2803360-supplementary.pdf]

Molecules

Aluminium 8-hydroxyquinolinate *N*-oxide as a precursor to heterometallic aluminium-lanthanide complexes

E. Gallo[1], L. Bellucci[1,2], S. Carlotto[2,3], G. Bottaro[2], L. Babetto[3], L. Giordano[1], F. Marchetti[1], S. Samaritani,[1] L. Armelao[3,4], L. Labella[1,2*]

[1] *Dipartimento di Chimica e Chimica Industriale and CIRCC, Università di Pisa, via Giuseppe Moruzzi 13, I-56124 Pisa*

[2] *CNR ICMATE and INSTM, presso Dipartimento di Scienze Chimiche, Università di Padova, via Marzolo 1,I-35131 Padova*

[3]*Dipartimento di Scienze Chimiche, Università di Padova, via Marzolo 1,I-35131 Padova*

[4]*Dipartimento di Scienze Chimiche e Tecnologie dei Materiali (DSCTM) Consiglio Nazionale delle Ricerche*

Piazzale A. Moro 7, 00185 Roma

[*] Correspondence: luca.labella@unipi.it (L.L.)

Supplementary Information

Table of contents

Characterization Data (List of Figures and Tables):

Figure S1: ATR-IR spectra (range 1700-650 cm^{-1}) of [Eu(hfac)$_3$Alq$_3$] (black), [Alq$_3$] (red) and [Eu(hfac)$_3$(H$_2$O)$_2$] (blue)

Figure S2: ATR-IR spectrum of 8-hydroxyquinoline *N*-oxide (HqNO)

Figure S3: ATR-IR spectrum of [Al(qNO)$_3$] (red) compared with [HqNO] (blue)

Figure S4: ^{1}H NMR of 8-hydroxyquinoline *N*-oxide (HqNO) in $CDCl_3$

Figura S5: ^{1}H NMR spectrum of [Al(qNO)$_3$] in anhydrous CD_2Cl_2.

Figure S6: ATR-IR spectra (1700-650 cm^{-1}) of [Eu(hfac)$_3$Al(qNO)$_3$] (black), of [Al(qNO)$_3$] (red) and [HqNO] (blue)

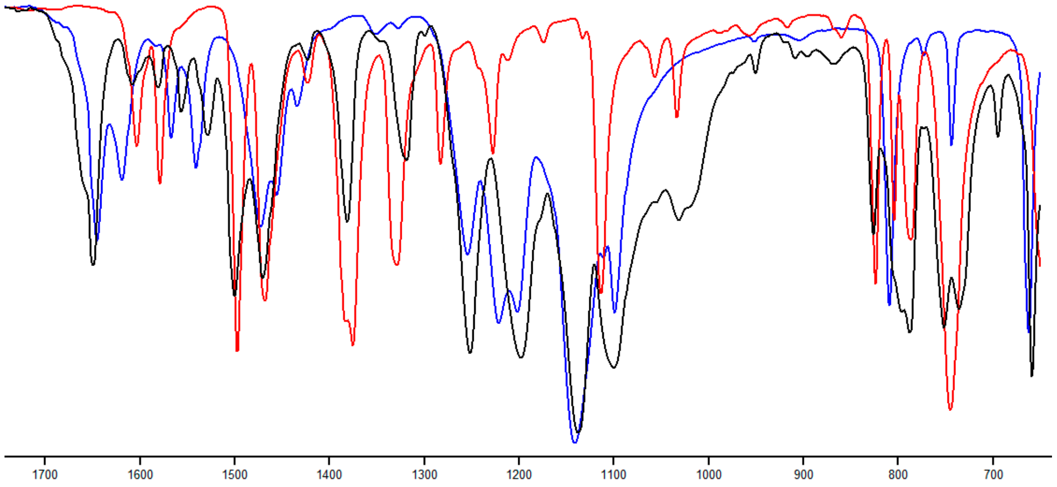

Figure S1: ATR-IR spectra (range 1700-650 cm^{-1}) of [Eu(hfac)$_3$Alq$_3$] (black), [Alq$_3$] (red) and [Eu(hfac)$_3$(H$_2$O)$_2$] (blue).

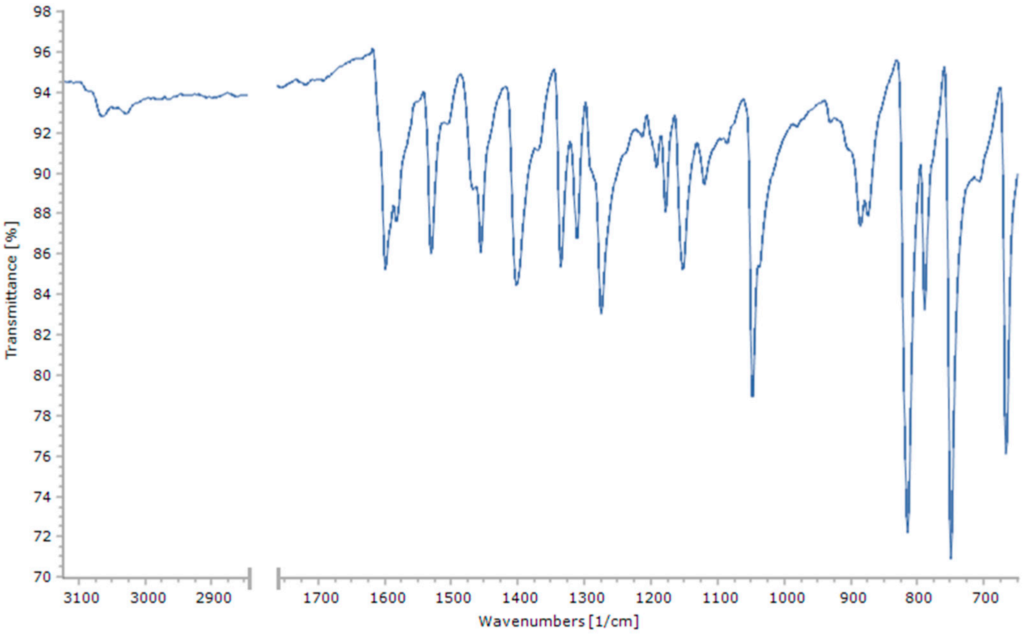

Figure S2: ATR-IR spectrum of 8-hydroxyquinoline *N*-oxide (HqNO).

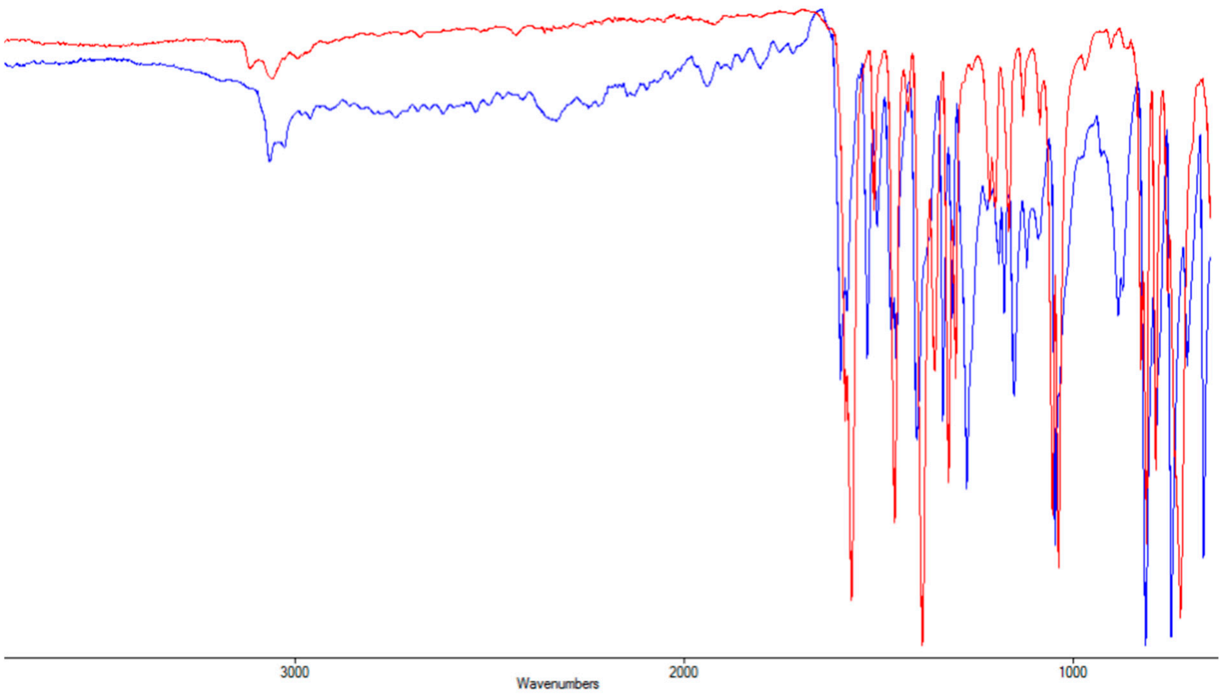

Figure S3: ATR-IR spectrum of $[Al(qNO)_3]$ (red) compared with [HqNO] (blue).

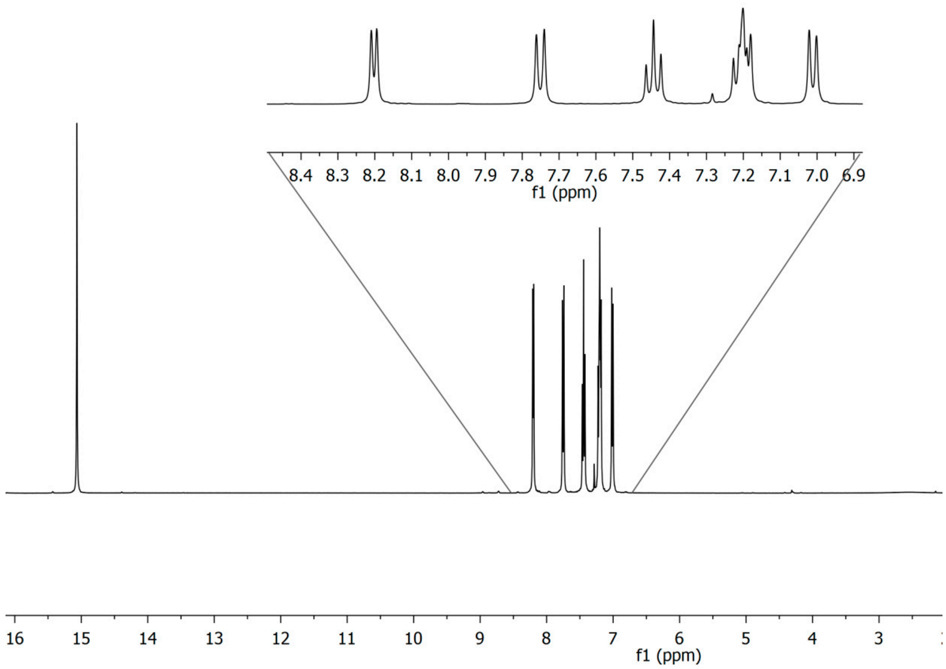

Figure S4: ^{1}H NMR of 8-hydroxyquinoline *N*-oxide (HqNO) in $CDCl_3$.

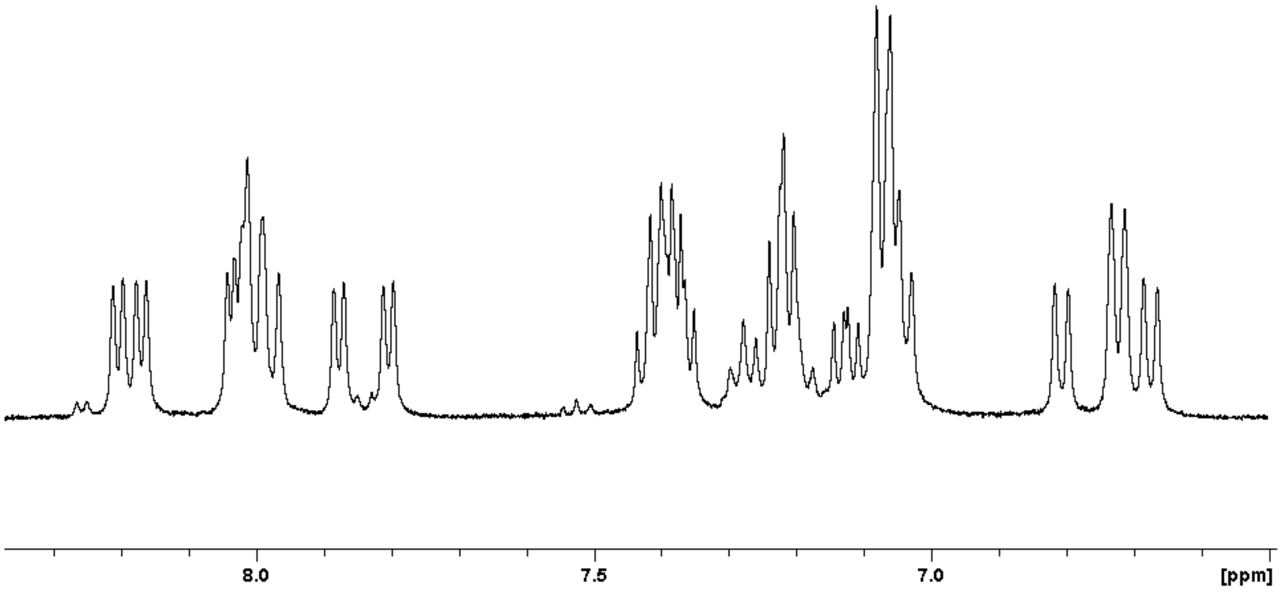

Figure S5: ^{1}H NMR spectrum of [Al(qNO)$_3$] in anhydrous CD_2Cl_2.

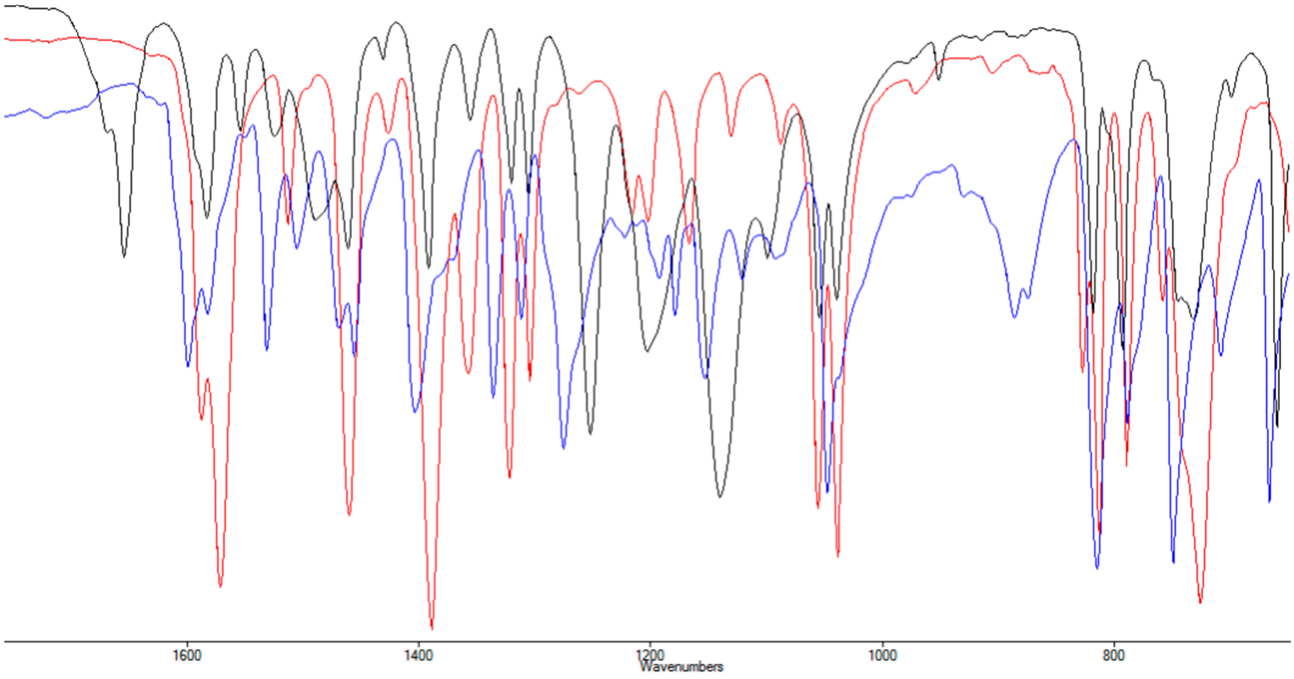

Figure S6: ATR-IR spectra (1700-650 cm^{-1}) of [Eu(hfac)$_3$Al(qNO)$_3$] (black), of [Al(qNO)$_3$] (red) and [HqNO] (blue).
